# Survey on antimicrobial resistance patterns in *Vibrio vulnificus* and *Vibrio cholerae* non-O1/non-O139 in Germany reveals carbapenemase-producing *Vibrio cholerae* in coastal waters

**DOI:** 10.3389/fmicb.2015.01179

**Published:** 2015-10-28

**Authors:** Nadja Bier, Keike Schwartz, Beatriz Guerra, Eckhard Strauch

**Affiliations:** Department of Biological Safety, Federal Institute for Risk AssessmentBerlin, Germany

**Keywords:** antimicrobial resistance pattern, Baltic Sea, North Sea, carbapenemase, disk diffusion, broth microdilution

## Abstract

An increase in the occurrence of potentially pathogenic *Vibrio* species is expected for waters in Northern Europe as a consequence of global warming. In this context, a higher incidence of *Vibrio* infections is predicted for the future and forecasts suggest that people visiting and living at the Baltic Sea are at particular risk. This study aimed to investigate antimicrobial resistance patterns among *Vibrio vulnificus* and *Vibrio cholerae* non-O1/non-O139 isolates that could pose a public health risk. Antimicrobial susceptibility of 141 *V. vulnificus* and 184 *V. cholerae* non-O1/non-O139 strains isolated from German coastal waters (Baltic Sea and North Sea) as well as from patients and retail seafood was assessed by broth microdilution and disk diffusion. Both species were susceptible to most of the agents tested (12 subclasses) and no multidrug-resistance was observed. Among *V. vulnificus* isolates, non-susceptibility was exclusively found toward aminoglycosides. In case of *V. cholerae*, a noticeable proportion of strains was non-susceptible to aminopenicillins and aminoglycosides. In addition, resistance toward carbapenems, quinolones, and folate pathway inhibitors was sporadically observed. Biochemical testing indicated the production of carbapenemases with unusual substrate specificity in four environmental *V. cholerae* strains. Most antimicrobial agents recommended for treatment of *V. vulnificus* and *V. cholerae* non-O1/non-O139 infections were found to be effective *in vitro*. However, the occurrence of putative carbapenemase producing *V. cholerae* in German coastal waters is of concern and highlights the need for systematic monitoring of antimicrobial susceptibility in potentially pathogenic *Vibrio* spp. in Europe.

## Introduction

The family *Vibrionaceae* within the class of Gammaproteobacteria comprises eight genera of Gram-negative, facultative anaerobic, straight, or curved rods that are mostly oxidase-positive, halophilic, and motile (Farmer and Janda, 2004). Members of this family are ubiquitously distributed in aquatic ecosystems worldwide. They can be found as free-living bacteria and as commensals of aquatic organisms and play an important role in nutrient cycling of natural aquatic habitats. Due to their metabolic diversity and their adaptive abilities to changing environmental conditions, a seasonal, and geographical variability of total *Vibrio* populations is observed in response to climatic influences and seawater circulations (Mansergh and Zehr, 2014).

Among the *Vibrionaceae*, a number of important human pathogenic bacteria have been identified that can cause gastrointestinal infections, wound infections or septicemia. *Vibrio cholerae, Vibrio vulnificus*, and *Vibrio parahaemolyticus* are considered as the most clinically relevant human pathogens within the genus *Vibrio* (Daniels and Shafaie, 2000). The latter species is widely disseminated in estuarine, marine, and coastal so surroundings and the leading cause of human intestinal infections after consumption of raw and undercooked seafood (Letchumanan et al., 2014). *V. cholerae* and *V. vulnificus* are also part of the microbial community in coastal or estuarine aquatic ecosystems with moderate salinities (Thompson and Polz, 2006).

*V. cholerae* is a well-known human pathogen consisting of more than 200 serogroups (Kaper et al., 1995; Lutz et al., 2013). Toxigenic *V. cholerae* of the O1 or O139 serogroup are the causative agents of cholera, an endemic disease in many Asian and African countries with symptoms of severe watery diarrhea, vomiting, and dehydration. All other serogroups designated as *V. cholerae* non-O1/non-O139 have also been linked to sporadically occurring human infections ranging from extraintestinal wound or ear infections (Huhulescu et al., 2007) to relatively mild or sometimes severe gastroenteritis (Tobin-D'Angelo et al., 2008), whereby smaller diarrheal outbreaks were also reported (Luo et al., 2013). Additionally, rarely occurring bacteremia has been described with mortality rates up to 61.5% (Petsaris et al., 2010). Since 2000, around 40 cases of *V. cholerae* non-O1/non-O139 infections in the United States have been reported to the CDC annually^1^. *V. cholerae* infections contracted in Germany were mainly ear or wound infections caused by non-toxigenic non-O1/non-O139 strains that were acquired through contact to seawater (Huehn et al., 2014). Due to the rare occurrence of *V. cholerae* non-O1/non-O139 infections, there are no official recommendations on antibiotic therapy (Petsaris et al., 2010). However, in case of bacteremia an early administration of antibiotic therapy can prevent a fatal outcome. Several case studies on *V. cholerae* non-O1/non-O139 bacteremia and wound infection exist, where fluoroquinolones and third-generation cephalosporins have been used (Huhulescu et al., 2007; Petsaris et al., 2010). But also treatment with ampicillin or last-line carbapenems has been described (Feghali and Adib, 2011; Lu et al., 2014).

*V. vulnificus* is known as a highly virulent pathogen. Although infections occur only sporadically, they can rapidly progress to septicemia, especially in persons with predisposing risk factors (e.g., immunocompromising conditions or chronic liver diseases resulting in elevated serum iron levels; Oliver, 2006). Foodborne infections can either result in a relatively mild gastroenteritis or in primary septicemia with mortality rates of 61% (Shapiro et al., 1998; Oliver, 2006). A second infection route for *V. vulnificus* is through open wounds exposed to seawater. Due to the high multiplication rate of the pathogen, wound infections may quickly progress to necrotizing fasciitis, which often makes surgical debridement or amputation necessary (Daniels and Shafaie, 2000). Delayed treatment promotes progression to secondary septicemia with mortality rates about seventeen per cent (Shapiro et al., 1998; Daniels and Shafaie, 2000). Surgical interventions should be considered early to prevent a fatal outcome as poor blood perfusion in necrotic tissue can impede the achievement of effective concentrations of antimicrobial agents (Chen et al., 2012). However, to avoid septicemia and a further distribution of the pathogen additional antibiotic therapy is indispensable and should be administered as early as possible. Due to the fast progression of *V. vulnificus* infections, the presence of antimicrobial resistance preventing an effective therapy can be fatal for the patient. A combination of a tetracycline with a third-generation cephalosporin or single-agent therapy with fluoroquinolones is recommended by the CDC^2^, while trimethoprim-sulfamethoxazole in combination with an aminoglycoside is proposed for the treatment of pregnant women and children.

In the U.S., 95% of all seafood-related deaths can be attributed to *V. vulnificus*, whereas infections in Germany were almost exclusively wound infections occurring after contact to seawater (Oliver, 2006; Huehn et al., 2014). So far, *Vibrio* infections in Germany occur only sporadically but incidences peaked after extreme heatwaves (Huehn et al., 2014). Due to impacts of climate change, a rise in the occurrence of *V. vulnificus* and *V. cholerae* is predicted for European waters (Baker-Austin et al., 2012). Changing demography is expected to further contribute to higher incidences of *Vibrio* infections (Baker-Austin et al., 2012). In view of these forecasts and the potential severity of infections, an investigation on antimicrobial susceptibility of *Vibrio* spp. is demanded to provide guidance for medical intervention, but also for epidemiological purposes. For this reason, our study aimed to assess antimicrobial resistance prevalence among *V. vulnificus* and *V. cholerae* non-O1/non-O139 posing a public health risk for the population. Environmental isolates were obtained from German coastal and estuarine waters of the open North Sea and the intracontinental Baltic Sea. In addition, we also included isolates from clinical sources and retail seafood for comparison and to give a more comprehensive overview of antimicrobial resistance patterns of these two species in Germany. To our knowledge, this is the first study examining antimicrobial susceptibility of *V. vulnificus* and *V. cholerae* non-O1/non-O139 in Northern Europe on a large scale.

## Materials and methods

### Bacterial strains

The strains used in this study are summarized in Table 1 and listed in detail in Supplementary Tables S1, S2. Antimicrobial susceptibilities were determined for a total of 325 bacterial strains, including 141 isolates of *V. vulnificus* (19 clinical, 122 environmental) and 184 isolates of *V. cholerae* non-O1/non-O139 (18 clinical, 131 environmental, 35 retail). The majority of environmental strains were isolated by health authorities during the German research programs KLIWAS^3^ and VibrioNet^4^ between 2004 and 2014. Water and sediment samples were mostly collected at bathing sites along the Baltic Sea and North Sea coastline as well as within the estuaries of the rivers Ems and Weser (Böer et al., 2012). Environmental isolates from bivalve mollusks were obtained from coastal areas of the North Sea. Isolates from retail samples were collected by health authorities of Germany and sent to the National Reference Laboratory for Monitoring Bacteriological Contamination of Bivalve Mollusks of the Federal Institute for Risk Assessment (BfR), Germany. Clinical *V. vulnificus* and *V. cholerae* non-O1/non-O139 isolates were characterized in previous studies (Bier et al., 2013; Schirmeister et al., 2014).

**Table 1:** **Origin and source of *V. cholerae* non-O1/non-O139 (*n* = 184) and *V. vulnificus* (*n* = 141) strains included in the study**.

**Origin**	**Geographical origin**	**Source**	**Source code**	**No. of strains**
***V. cholerae***				
Environmental (E) (2009–2014; *n* = 131)	Baltic Sea (BS) (*n* = 79)	Seawater (sw)	E-BS-sw	54
		Sediment (sd)	E-BS-sd	4
		Seawater/sediment (sw/sd)	E-BS-sw/sd	21
	North Sea (NS) (*n* = 52)	Bivalve mollusks (bm)	E-NS-bm	26
		Seawater (sw)	E-NS-sw	12
		Seawater/sediment (sw/sd)	E-NS-sw/sd	14
Clinical (C) (1995–2012; *n* = 18)	Travel-associated (ta) (*n* = 7)	Extraintestinal (ext)	C-ta-ext	1
		Intestinal (int)	C-ta-int	6
	Germany/Austria (G/A) (*n* = 11)	Extraintestinal (ext)	C-G/A-ext	9
		Intestinal (int)	C-G/A-int	2
Retail (R) (2008–2014; *n* = 35)	Germany (G) (*n* = 35)	Bivalve mollusks (bm)	R-G-bm	2
		Crustacean (cr)	R-G-cr	26
		Fish (fi)	R-G-fi	7
***V. vulnificus***				
Environmental (E) (2004–2012; *n* = 122)	Baltic Sea (BS) (*n* = 70)	Seawater (sw)	E-BS-sw	46
		Sediment (sd)	E-BS-sd	24
	North Sea (NS) (*n* = 52)	Seawater (sw)	E-NS-sw	29
		Sediment (sd)	E-NS-sd	21
		Bivalve mollusks (bm)	E-NS-bm	2
Clinical (C) (1994–2011; *n* = 19)	Denmark (D)	Extraintestinal (ext)	C-D-ext	14
	Germany (G)	Extraintestinal (ext)	C-G-ext	5

### DNA extraction

DNA extraction was performed with two methods that are equally applicable for *Vibrio* species. Genomic DNA of *V. vulnificus* isolates was extracted from 1 ml of an overnight culture using the RTP Bacteria DNA Kit according to the manufacturer's protocol (STRATEC Biomedical AG, Birkenfeld, Germany).

Genomic DNA of *V. cholerae* strains was extracted using a boiling method: 1.5 ml of an overnight culture were centrifuged at 14,000 g for 4 min. The cell pellet was suspended in 300 μl TE buffer (10 mM Tris-HCl, 0.5 mM EDTA, pH 8), boiled for 10 min at 95°C, and subsequently cooled on ice. After centrifugation at 14,000 g for 2 min, a 200 μl aliquot of the supernatant was transferred to a new sterile tube. DNA preparations were stored at −20°C.

### Species confirmation

Species confirmation of all *V. vulnificus* and *V. cholerae* strains was carried out by species-specific *toxR* PCR amplification as previously described (Bauer and Røervik, 2007) and in parallel by MALDI-TOF MS analysis. MALDI-TOF MS analysis was performed using a Microflex LT system mass spectrometer (Bruker Daltonik, Bremen, Germany) following the manufacturer's settings. MALDI spectra were obtained by the direct transfer method according to the manufacturer's protocol as previously described (Schirmeister et al., 2014).

### Characterization of *V. cholerae* isolates

*V. cholerae* isolates were characterized and subtyped via multiplex PCR targeting *rfb* sequences specific for O1 and O139 serogroups, *toxR*, and *ctxA*. PCR amplification was performed in a final volume of 25 μl with 1x PCR buffer (3 mM MgCl_2_), 0.2 mM of each deoxynucleoside triphosphate, 0.5 μM of O1 *rfb* primers, 0.125 μM of O139 *rfb, toxR*, and *ctxA* primers, 1.5 U DreamTaq DNA polymerase (Thermo Fisher Scientific Biosciences GmbH, St. Leon-Rot, Germany), and 2 μl of genomic DNA. After an initial denaturation step at 94°C for 4 min, the cycling conditions were the following: 30 cycles at 94°C for 30 s, 59°C for 30 s, 72°C for 30 s, followed by a final extension step at 72°C for 5 min.

### Antimicrobial susceptibility testing

Antimicrobial susceptibility to the following 13 antimicrobial agents was determined by broth microdilution according to the guidelines of the Clinical and Laboratory Standards Institute (CLSI, 2012a) using custom-defined microtiter plates (EUMVS2, Trek Diagnostic Systems, East Grinstead, United Kingdom): ampicillin, ceftazidime, cefotaxime, chloramphenicol, ciprofloxacin, colistin, florfenicol, gentamicin, kanamycin, nalidixic acid, streptomycin, tetracycline, and trimethoprim. Test ranges are shown in Supplementary Table S3. Additionally, all isolates were tested for their susceptibility to amoxicillin-clavulanic acid (20/10 μg), cefepime (30 μg), imipenem (10 μg), levofloxacin (5 μg), meropenem (10 μg), and sulfamethoxazole-trimethoprim (23.75/1.25 μg) by the disk diffusion method, according to the guidelines of the CLSI using commercially available disks (Oxoid GmbH, Wesel, Germany; CLSI, 2012b).

Strains showing non-susceptibility to imipenem (zone diameter ≤ 19) were tested against an additional panel of β-lactams by broth microdilution (imipenem, ertapenem, cefepime, cefoxitin, temocillin; EUVSEC2, Trek Diagnostic Systems) or disk diffusion (aztreonam, 30 μg).

Following the guidelines of the CLSI, tests were performed with Mueller-Hinton agar and cation-adjusted Mueller-Hinton broth without supplementation of additional sodium chloride (CLSI, 2010a). *Escherichia coli* ATCC 25922 was used for quality assurance. Minimal inhibitory concentration (MIC) values and inhibition zone diameters of all strains are listed in Supplementary Tables S1, S2. Results were interpreted using the criteria summarized in Supplementary Table S3. In general, results were interpreted according to CLSI clinical breakpoints specific for *Vibrio* spp. (CLSI, 2010a), which derived from breakpoints for *Enterobacteriaceae* (CLSI, 2010b). In cases where CLSI breakpoints for *Vibrio* spp. were obsolete or not available, the latest CLSI breakpoints for *Enterobacteriaceae* were used: document M100-S25 for aztreonam, cefepime, ertapenem, gentamicin, kanamycin, imipenem, meropenem, nalidixic acid, and trimethoprim (CLSI, 2015); document Vet01-S2 for florfenicol (CLSI, 2013). Other interpretive criteria were used for colistin (EUCAST clinical breakpoints for *Enterobacteriaceae*^5^; EUCAST, 2015), temocillin (BSAC interpretive criteria for systemic infections; Andrews, 2009), and streptomycin (based on different studies of *Vibrio* spp. and *E. coli*; National Food Institute, 2013; Shaw et al., 2014), as no CLSI breakpoints were available.

### Molecular investigation of resistance determinants

PCR amplification was conducted to detect specific antimicrobial resistance determinants depending on the phenotype found. Non-susceptible isolates were screened for genes mediating resistance to streptomycin (*aadA1, aadA2*, and *strA/B*) and β-lactams (*bla*_PSE−1_, *bla*_OXA−1−like_, *bla*_TEM−1−like_) that are widespread in *Enterobacteriaceae* and other Gram-negative bacteria. Specifically imipenem-resistant strains (zone diameter ≤ 19) were tested for the presence of several carbapenemase and AmpC β-lactamase encoding genes. Presence of class 1 integrons was investigated by PCR amplification of the corresponding integrase gene *intI1* in all β-lactam and streptomycin non-susceptible strains.

Standard PCR reactions were performed using a Mastercycler EP gradient (Eppendorf, Hamburg, Germany) in a volume of 25 μl with 1x PCR buffer (2 mM MgCl_2_), 0.2 mM of each deoxynucleoside triphosphate (dNTP), 0.2 μM of each primer, 1.5 U DreamTaq DNA polymerase, and 2 μl of genomic DNA. After an initial denaturation step at 94°C for 4 min, the cycling conditions were the following: 30 cycles of denaturation at 94°C for 30 s, primer annealing for 30 s, and extension at 72 °C for 1 min per kb, followed by a final extension step at 72°C for 10 min.

All primer pairs, target genes, corresponding annealing temperatures, and amplicon sizes are listed in Supplementary Table S4. Enterobacterial strains carrying class 1 integrons and investigated resistance determinants (*aadA1, aadA2, bla*_PSE−1_, *bla*_OXA−1_, *bla*_TEM−1_, *bla*_NDM−1_*, bla*_IMP−1_*, bla*_VIM−2_*, bla*_KPC−3_, *bla*_OXA−48_, *bla*_ACC−1_, *bla*_CMY−1_, *bla*_CMY−2_, *bla*_DHA−1_, *bla*_ACT−1_, *bla*_FOX−1_, *strA*, and *strB*), as well as one *V. cholerae* non-O1/non-O139 isolate carrying a class 1 integron with the *aadA1* gene were used as positive controls. Susceptible *V. cholerae* and *V. vulnificus* strains were included as negative controls.

All streptomycin resistant *V. cholerae* (VN-3469, VN-5095, VN-10191, and VN-10192) and *V. vulnificus* (VN-0098, VN-0100, VN-0125, VN-0129) isolates were further examined for mutations in the *rpsL* gene encoding ribosomal protein S12. Streptomycin susceptible (*V. cholerae*: VN-0298, VN-2997, VN-3955, VN-4226, and *V. vulnificus*: VN-0096, VN-0274, VN-3368) and intermediate resistant strains (*V. cholerae*: VN-3944, VN-4261, and *V. vulnificus*: VN-3418, VN-3981, VN-10121) were included as controls. For specific amplification and sequencing of the whole *rpsL* gene in *V. cholerae* and *V. vulnificus*, two primer pairs Vc-rpsL-F/Vc-rpsL-R and Vv-rpsL-F/Vv-rpsL-R were designed based on published genome sequences (*V. cholerae* strains NIH41, N16961, O395, and *V. vulnificus* strains CMCP6, YJ016, MO6-24/O). Purification of PCR products was performed using the MSB® Spin PCRapace Kit (STRATEC Biomedical AG, Berlin, Germany). Sequencing was conducted on both strands through sequencing service (Eurofins MWG GmbH, Ebersberg, Germany). Electropherograms were assembled and trimmed using SeqMan Pro (v12; DNASTAR Lasergene, Madison, Wisconsin). Sequences were analyzed and compared to the sequences of reference and control strains using Accelrys Gene (v2.5, Accelrys Inc., San Diego, California).

### Test for carbapenemase activity: Carba NP test II/Blue-CARBA test

Strains non-susceptible to imipenem (zone diameter ≤ 19) were grown overnight at 37°C on chromID™ CARBA (bioMérieux, Nürtingen, Germany). Bacterial colonies were subsequently tested for carbapenemase activity with the improved Carba NP test II (Dortet et al., 2014) using 12 g/L imipenem/cilastatin (Zienam®, MSD SHARP, and DOHME GMBH, Haar, Germany) and two calibrated loops (10 μl) as bacterial inoculum to increase enzyme quantity. The Blue-CARBA test was performed as described (Pires et al., 2013) and in addition analogously to the Carba NP test II with two loops of bacterial colonies in 200 μl of the test solution in microcentrifuge tubes and supplementation of tazobactam or EDTA to inhibit class A or metallo-carbapenemases, respectively. Two *E. coli* strains positive for NDM-1 and KPC-2, respectively, as well as a KPC-3-positive *Klebsiella pneumoniae* strain served as positive controls.

### Statistical analyses

Descriptive statistics were used to analyze resistance prevalence to different antimicrobial agents (Table 2). Chi-square test for independence was applied with 2 × 2 contingency tables to test if observed differences displayed in Table 3 were statistically significant (*P*-values ≤ 0.05). MIC_50_ and MIC_90_ were defined as the concentration at which growth of 50 and 90% of the isolates was inhibited, respectively.

**Table 2:** **Susceptibility vs. resistance occurrence (%) found among *V. cholerae* non-O1/non-O139 isolates from different origins^*a*^**.

**Antimicrobial agent**	**Total (*n* = 184)**			**Retail (*n* = 35)**			**Clinical (*n* = 18)**			**Environmental (*n* = 131)**			**North Sea (*n* = 52)**			**Baltic Sea (*n* = 79)**		
	**S**	**I**	**R**	**S**	**I**	**R**	**S**	**I**	**R**	**S**	**I**	**R**	**S**	**I**	**R**	**S**	**I**	**R**
Amoxicillin/clavulanic acid	98	2	0	100	0	0	100	0	0	97	3	0	94	6	0	99	1	0
Ampicillin	89	0	11	89	0	11	83	0	17	90	0	10	85	0	15	94	0	6
Imipenem	97	1	2	100	0	0	100	0	0	95	2	3	94	0	6	96	3	1
Meropenem	98	2	< 1	100	0	0	100	0	0	97	2	1	94	4	2	99	1	0
Nalidixic acid	99	0	1	100	0	0	89	0	11	100	0	0	100	0	0	100	0	0
Streptomycin	78	20	2	86	11	3	83	17	0	75	23	2	65	29	6	81	19	0
Trimethoprim	99	0	1	97	0	3	100	0	0	100	0	0	100	0	0	100	0	0

*S, susceptible; I, intermediate resistant; R, resistant*.

a *All strains were susceptible to ceftazidime, chloramphenicol, ciprofloxacin, cefotaxime, cefepime, florfenicol, gentamicin, kanamycin, levofloxacin, trimethoprim/sulfamethoxazole and tetracycline*.

**Table 3:** **Overall resistance occurrence in *V. cholerae* and *V. vulnificus* isolates with respect to different origins**.

	**Retail**	**Clinical**	**Environmental**	**North Sea**	**Baltic Sea**
*V. cholerae*	(*n* = 35)	(*n* = 18)	(*n* = 131)	(*n* = 52)	(*n* = 79)
Strains susceptible to all antimicrobial agents	27 (77%)	10 (56%)	86 (66%)	28 (54%)	58 (73%)
Non-susceptible strains	8 (23%)	8 (44%)	45 (34%)	24(46%)	21 (27%)
*V. vulnificus*		(*n* = 19)	(*n* = 122)	(*n* = 52)	(*n* = 70)
Strains susceptible to all antimicrobial agents		8 (42%)	72 (59%)	28 (54%)	44 (63%)
Non-susceptible strains		11 (58%)	50 (41 %)	24 (46 %)	26 (37 %)

## Results

### Antimicrobial susceptibility of clinical and environmental *V. vulnificus* isolates

Fifty-seven per cent of all examined *V. vulnificus* isolates showed susceptibility to all antimicrobial agents tested (with the exception of colistin). All 141 *V. vulnificus* isolates, regardless of their origin were susceptible to quinolones, fluoroquinolones, phenicols, tetracyclines, folate pathway inhibitors, aminopenicillins with or without β-lactamase inhibitors, carbapenems, and third- and fourth- generation cephalosporins. The clinically relevant agents cefotaxime, ceftazidime, and tetracycline were among the most effective antimicrobial agents *in vitro* as they showed MIC_90_ values identical to the lowest concentration tested (Table 4). Non-susceptibility was exclusively observed toward aminoglycosides with 40 and 3% of all strains showing intermediate resistance (MIC 32 mg/L) and resistance (MIC 64 mg/L) to streptomycin, respectively. One clinical isolate was intermediate resistant to kanamycin (MIC 32 mg/L) while all strains were susceptible to gentamicin. The percentage of non-susceptible strains with respect to different origins is shown in Table 3. No significant difference was observed between clinical (*n* = 19) and environmental (*n* = 122) isolates, nor was there a significant difference between isolates from the Baltic Sea and the North Sea (*p* > 0.05, χ^2^). None of the examined gene determinants encoding streptomycin resistance (*aadA1, aadA2, strA/B*), nor class 1 integrons were detected in streptomycin non-susceptible *V. vulnificus* isolates. Sequence analysis of the *rpsL* gene revealed that all four streptomycin resistant *V. vulnificus* isolates (VN-0098, VN-0100, VN-0125, VN-0129) as well as two susceptible (VN-0274, VN-3368) and two intermediate resistant isolates (VN-3918, VN-10121) carried one silent point mutation A-291-T compared to the three reference strains (CMCP6, YJ016, and MO6-24/O). An additional silent mutation C-351-T within the *rpsL* gene was observed in strain VN-0100.

**Table 4:** **Antimicrobial MIC distributions for the *V. cholerae* and *V. vulnificus* isolates tested**.

**Antimicrobial agent**	**Test range (mg/L)**	**Breakpoints^*a*^ (mg/L)**			**MIC (mg/L) distribution for *V. vulnificus* (*n* = 141)**			**MIC (mg/L) distribution for *V. cholerae* (*n* = 184)**		
		***S***	***I***	***R***	**MIC_50_**	**MIC_90_**	**Range**	**MIC_50_**	**MIC_90_**	**Range**
Ampicillin	0.5–32	≤ 8	16	≥32	1	2	≤ 0.5–2	2	8	≤ 0.5–>32
Cefotaxime	0.06–4	≤ 1	2	≥4	≤ 0.06	≤ 0.06	≤ 0.06–0.12	≤ 0.06	≤ 0.06	≤ 0.06–0.12
Ceftazidime	0.25–16	≤ 4	8	≥16	≤ 0.25	≤ 0.25	≤ 0.25–0.5	≤ 0.25	≤ 0.25	≤ 0.25–0.5
Chloramphenicol	2–64	≤ 8	16	≥32	≤ 2	≤ 2	≤ 2	≤ 2	≤ 2	≤ 2
Ciprofloxacin	0.008–8	≤ 1	2	≥4	0.015	0.03	≤ 0.008–0.06	≤ 0.008	≤ 0.008	≤ 0.008–0.5
Florfenicol	2–64	≤ 4	8	≥16	≤ 2	≤ 2	≤ 2	≤ 2	≤ 2	≤ 2
Gentamicin	0.25–32	≤ 4	8	≥16	2	2	0.5–4	1	2	≤ 0.25–4
Kanamycin	4–128	≤ 16	32	≥64	8	16	≤ 4–32	≤ 4	8	≤ 4–16
Nalidixic acid	4–64	≤ 16		≥32	≤ 4	≤ 4	≤ 4–8	≤ 4	≤ 4	≤ 4–>64
Streptomycin	2–128	≤ 16	32	≥64	16	32	4–64	16	32	8–64
Tetracycline	1–64	≤ 4	8	≥16	≤ 1	≤ 1	≤ 1–2	≤ 1	≤ 1	≤ 1
Trimethoprim	0.5–32	≤ 8		≥16	1	1	≤ 0.5–4	≤ 0.5	1	≤ 0.5–>32

*S, susceptible; I, intermediate resistant; R, resistant*.

a *Criteria used for interpretation and corresponding references are given in Supplementary Table S3*.

### Antimicrobial susceptibility of *V. cholerae* non-O1/non-O139 isolated from clinical, environmental and seafood samples

All 184 isolates investigated in this study were confirmed to be non-toxigenic *V. cholerae* non-O1/non-O139 isolates. The majority of isolates (67%) were susceptible to all antimicrobial agents tested (with the exception of colistin). Eighteen per cent of the strains showed intermediate resistance to one or two antimicrobial agents (mostly to streptomycin) and the remaining strains (15%) showed full resistance to at least one antimicrobial agent. None of the *V. cholerae* isolates showed multidrug-resistance, defined as resistance to three or more classes of antimicrobial agents (Chen et al., 2010). Resistance profiles are shown in Supplementary Table S2, while resistance occurrence is given in Table 2.

As observed among *V. vulnificus*, all *V. cholerae* strains were susceptible to ciprofloxacin, chloramphenicol, florfenicol, cefotaxime, sulfamethoxazole-trimethoprim, levofloxacin, ceftazidime, cefepime, gentamicin, kanamycin, and tetracycline. Additionally, 98% of the isolates were susceptible to amoxicillin/clavulanic acid. The most effective clinically relevant agents *in vitro* were ciprofloxacin, cefotaxime, ceftazidime, and tetracycline as they showed MIC_90_ values identical to the lowest concentration tested (Table 4).

Similar to the *V. vulnificus* isolates, a small proportion of *V. cholerae* strains showed resistance to streptomycin (2%), while 20% of the strains were intermediate resistant. In contrast to *V. vulnificus*, the most frequent antimicrobial resistance found among all *V. cholerae* isolates was resistance to ampicillin (11%). Resistance to nalidixic acid and trimethoprim was rarely observed in two clinical isolates and in one isolate from seafood. Non-susceptibility to the carbapenems imipenem and meropenem was observed in 5 and 3% of the environmental isolates, respectively.

Clinical strains showed the highest percentage of non-susceptible strains (44%), followed by environmental strains (34%) and by strains isolated from retail seafood (23%) (Table 3). However, statistical analysis revealed that the observed differences to environmental isolates are not significant (*p* > 0.05, χ^2^). Comparison between the geographical origin of environmental strains revealed that the percentages of strains non-susceptible to streptomycin, ampicillin, amoxicillin/clavulanic acid, meropenem, and imipenem were higher in the North Sea compared to the Baltic Sea (Table 2). The higher occurrence of non-susceptible *V. cholerae* strains in the North Sea (Table 3) was statistically significant (χ2 = 5.327, d.f. = 1, *p* < 0.05).

Analysis of MIC distributions revealed that susceptibilities of the two *Vibrio* species were rather similar (Table 4). Differences were exclusively observed for kanamycin and ciprofloxacin, where MIC_90_ values of *V. cholerae* were two and four times lower and in case of ampicillin four times higher than those for *V. vulnificus*.

Susceptibility to colistin was excluded from any statistical analysis and tables, since *V. vulnificus* and *V. cholerae* possess an intrinsic resistance to colistin, which is used for selective growth on cellobiose-polymyxin B-colistin agar (Massad and Oliver, 1987). However, five *V. cholerae* strains were highly susceptible to colistin (MICs ≤ 2 mg/L) and would therefore fail to grow on this selective agar.

Neither class 1 integrons, nor gene determinants encoding streptomycin resistance (*aadA1, aadA2, strA/B*) were detected among non-susceptible *V. cholerae* isolates. Sequence analysis of the *rpsL* gene revealed three silent point mutations C-198-T, A-251-T, and G-360-A in two streptomycin resistant *V. cholerae* isolates (VN-10191, VN-10192) as well as within a susceptible isolate (VN-4226). In addition, PCR amplifications to detect β-lactamase genes (*bla*_PSE−1_, *bla*_OXA−1−like_, *bla*_TEM−1−like_) were negative in all tested *V. cholerae* isolates.

### Examination of carbapenem non-susceptible *V. cholerae* isolates

Among the 131 environmental *V. cholerae* non-O1/non-O139 isolates analyzed, resistance to the carbapenem imipenem (zone diameter ≤ 19) was observed in four strains (VN-2808, VN-2825, VN-2923, and VN-2997). These strains additionally showed resistance to ampicillin, intermediate resistance to amoxicillin-clavulanic acid, as well as intermediate or full resistance to meropenem (Table 5). In contrast, they were susceptible to the third- and fourth-generation cephalosporins ceftazidime, cefotaxime, and cefepime. Further characterization revealed resistance to aztreonam and ertapenem, while the strains were susceptible to temocillin and intermediate resistant to cefoxitin. The four strains grew on chromID™ CARBA agar, while growth was inhibited on chromID™ OXA-48 (bioMérieux GmbH, Nürtingen, Germany), indicating the expression of carbapenem-hydrolyzing enzymes other than OXA-48 type carbapenemases. To further investigate the presence of carbapenemases, Blue-CARBA and Carba NP II tests were conducted on these strains. Imipenem-hydrolyzing activity was detected in intact cells and crude cell extracts of each of the four strains. This activity was inhibited by tazobactam but not by EDTA, suggesting the presence of Ambler class A carbapenemases rather than class B metallo-carbapenemases or class D OXA-carbapenemases (Dortet et al., 2012).

**Table 5:** **β-lactam MIC values and inhibition zone diameters found in the putative carbapenemase-producers and in carbapenem susceptible isolates selected as negative controls**.

	**Antimicrobial agent**				**Strain ID of *V. cholerae* isolates**						
		**Breakpoints^*d*^**			**Putative carbapenemase-producers**				**Carbapenem susceptible controls**		
		**S**	**I**	**R**	**VN-02997**	**VN-02825**	**VN-02923**	**VN-02808**	**VN-10145**	**VN-00301**	**VN-00161**
MIC values (mg/L)^a^	Ampicillin	≤ 8	16	≥32	>32	>32	>32	>32	2	2	2
	Cefepime	≤ 8	16	≥32	0.5	0.25	0.5	0.25	0.12	≤ 0.06	≤ 0.06
	Cefoxitin	≤ 8	16	≥32	16	16	16	16	4	4	4
	Cefotaxime	≤ 1	2	≥4	0.12	≤ 0.06	≤ 0.06	≤ 0.06	≤ 0.06	≤ 0.06	≤ 0.06
	Ceftazidime	≤ 4	8	≥16	0.5	0.5	≤ 0.25	≤ 0.25	≤ 0.25	≤ 0.25	≤ 0.25
	Ertapenem	≤ 0.5	1	≥2	2	>2	>2	2	0.12	0.03	0.06
	Imipenem	≤ 1	2	≥4	16	>16	>16	16	1	0.5	1
	Temocillin	≤ 8	-	>8	4	4	4	4	4	1	1
Inhibition zone diameter (mm)^b^	Amoxicillin/clavulanic acid	≥18	14–17	≤ 13	14	13.5	14	15	23	28	18
	Aztreonam	≥21	18–20	≤ 17	12	15	17	16	NA	NA	29
	Cefepime	≥25	19–24	≤ 18	28	26	28	28	34	40	30
	Ceftazidime	≥21	18–20	≤ 17	28	26	28	28	NA	NA	31
	Imipenem	≥23	20–22	≤ 19	14	15	16	15	30	36	26
	Meropenem	≥23	20–22	≤ 19	20	20	20	19	34	36	28
β-lactam resistance phenotype^c^					(AMC)- AMP-ATM- ETP-(FOX)- IPM-(MEM)	(AMC)- AMP-ATM- ETP-(FOX)- IPM-(MEM)	(AMC)- AMP-ATM- ETP-(FOX)- IPM-(MEM)	(AMC)- AMP-ATM- ETP-(FOX)- IPM-MEM	–	–	–

*MIC, minimal inhibitory concentration; S, susceptible; I, intermediate resistant; R, resistant; NA, not assessed; AMC, amoxicillin/clavulanic acid; AMP, ampicillin; ATM, aztreonam; ETP, ertapenem; FOX, cefoxitin; IPM, imipenem; MEM, meropenem*.

a *MIC values obtained by broth microdilution*.

b *Inhibition zone diameter obtained by disk diffusion*.

c *Results against non-β-lactams are not displayed; intermediate resistance is shown in brackets*.

d *Criteria used for interpretation with corresponding references are given in Supplementary Table S3*.

PCRs to identify genes encoding Ambler class A carbapenemases were performed. However, no products with expected sizes were observed using primers for detection of NMC-A, SME 1-3, IMI 1-3, or KPC 1-5. In the case of NMC-A, SME, and IMI, a general failure of PCR amplification cannot be excluded as no positive control strains were available.

In addition, the strains were negative for PCR-amplification of specific genes encoding Ambler class B metallo-carbapenemases (VIM 1-2, IMP, NDM-1), class D carbapenemase (OXA-48), and AmpC-β-lactamases (MOX 1-2, CMY 1-11, LAT 1-4, BIL-1, DHA 1-2, ACC, MIR-1T, ACT-1, FOX 1-5b) as well as of class 1 integrons.

## Discussion

### Prevalence of antimicrobial resistance in *V. vulnificus* and *V. cholerae* non-O1/non-O139

In this study, *V. vulnificus* isolates from German coastal waters as well as of clinical origin were susceptible to quinolones, fluoroquinolones, phenicols, tetracyclines, folate pathway inhibitors, aminopenicillins with or without β-lactamase inhibitors, carbapenems, and third- and fourth-generation cephalosporins. Non-susceptibility was exclusively observed toward aminoglycosides; predominantly streptomycin and sporadically kanamycin. Similar observations were made by Han et al. (2007), who reported total susceptibility with comparable MIC_90_ values to chloramphenicol, ampicillin, ceftazidime, cefotaxime, ciprofloxacin, gentamicin, and tetracycline among *V. vulnificus* isolates from oysters of the Louisiana Gulf coast, USA (Han et al., 2007). In a recent study of *V. vulnificus* isolates from the Chesapeake Bay, USA (Shaw et al., 2014), the highest percentage of resistance was also observed against streptomycin. However, a large percentage of intermediate resistant strains to chloramphenicol (78%) and sporadically non-susceptibility to β-lactams was also reported in that study (Shaw et al., 2014). Compared to our study, higher percentages of non-susceptible strains to ampicillin, tetracycline, nalidixic acid, trimethoprim, and especially to the aminoglycosides streptomycin and gentamicin were observed in a study of 151 *V. vulnificus* isolates from South Carolina, USA, while resistance to chloramphenicol and meropenem was under one per cent (Baker-Austin et al., 2009).

Among *V. cholerae* non-O1/non-O139 isolated from clinical, environmental, and seafood samples in Germany, no multidrug-resistance was observed and the majority (67%) of isolates were susceptible to all antimicrobial agents tested. Full resistance was most frequently found toward ampicillin (11%) and streptomycin (2%). In addition, a considerable proportion of isolates showed intermediate resistance to streptomycin (20%). Resistance to nalidixic acid and trimethoprim was only sporadically found in isolates from clinical and seafood samples, respectively. While numerous studies on antimicrobial resistance of toxigenic *V. cholerae* O1, O139 strains have been published, data on *V. cholerae* non-O1/non-O139 are less frequent. A recent study showed similar antimicrobial resistance patterns among environmental *V. cholerae* non-O1/non-O139 isolates from the Chesapeake Bay, USA (Ceccarelli et al., 2015). No multidrug-resistant isolates were detected and resistance to β-lactams was found in some isolates. In one large-scale study from India on antimicrobial susceptibility of *V. cholerae* non-O1/non-O139 isolates, the highest percentage of resistant strains was also seen for ampicillin (88%) and streptomycin (85%) though with a considerably higher frequency (Kumar et al., 2009). In contrast to our study, Kumar et al. (2009) reported a high prevalence of multidrug resistance and only a small percentage of strains were susceptible to all ten antimicrobial agents tested (12%).

Several PCR analyses were performed to reveal the underlying molecular mechanisms responsible for the observed non-susceptibility to streptomycin, ampicillin, and imipenem.

Resistance to streptomycin is often mediated by enzymatic inactivation through adenylation by aminoglycoside (3″) adenyltransferases (*aadA* genes) or through phosphorylation by aminoglycoside phosphotransferases (*strA*/*strB* genes; Shaw et al., 1993; Tsai et al., 2014). However, none of the *Vibrio* isolates was positive for amplification of *aadA1, aadA2*, or *strA/B* genes that are commonly found in *Enterobacteriaceae* and that have already been identified in *V. cholerae* (Hochhut et al., 2001; Sá et al., 2010; Yu et al., 2012). Ribosomal alterations resulting from mutations in the *rpsL* gene encoding ribosomal protein S12 can be another cause of streptomycin resistance (Shaw et al., 1993; Tsai et al., 2014). However, amino acid sequences of ribosomal protein S12 were identical in all investigated strains, irrespective of the streptomycin resistance phenotype. Observed single nucleotide polymorphisms within the *rpsL* gene of some resistant as well as of some susceptible isolates were silent mutations. This indicates that other streptomycin inactivating enzymes or other resistance mechanisms, such as decreased permeability or other ribosomal alterations (e.g., mutations in the *rrs* gene encoding the 16S ribosomal RNA) may be responsible for the observed phenotype (Shaw et al., 1993; Tsai et al., 2014).

Likewise, all β-lactam non-susceptible *Vibrio* isolates were negative for amplification of *bla*_PSE−1_, *bla*_OXA−1−like_, and *bla*_TEM−1−like_ genes encoding common β-lactamases in *Enterobacteriaceae* and other Gram-negative bacteria. This suggests that resistance may be encoded by other β-lactamase genes which are known to show a high diversity in Gram-negative bacteria. β-lactam resistance may also be mediated by other mechanisms such as reduced permeability, increased efflux, or target alterations (e.g., reduced affinity or increased amount of penicillin-binding protein (PBP); Foster, 1983).

### Carbapenemase producing *V. cholerae*

Antimicrobial susceptibility patterns as well as growth patterns on different selective media indicated the presence of a β-lactamase with carbapenem hydrolyzing activity in four environmental *V. cholerae* non-O1/non-O139 isolates from the Baltic Sea and the North Sea. The expression of carbapenemases was confirmed by positive Blue-CARBA and positive Carba NP II tests, which specifically detect imipenem hydrolyzing activity (Dortet et al., 2012; Pires et al., 2013). Inhibition of carbapenemase activity by tazobactam but not by EDTA suggested the presence of Ambler class A carbapenemases rather than class B metallo-carbapenemases or class D OXA-carbapenemases (Dortet et al., 2012). However, we found no evidence for the presence of specific genes encoding Ambler class A carbapenemases.

The four strains showed an exceptional resistance profile: They were non-susceptible to aminopenicillins, carbapenems, cefoxitin, and aztreonam and were only slightly inhibited by the β-lactamase inhibitor clavulanic acid. However, they were fully susceptible to third- and fourth-generation cephalosporins as well as to temocillin. The observed resistance to aminopenicillins coupled with susceptibility to extended spectrum cephalosporins may indicate the presence of an OXA-type carbapenemase (Ambler class D) reviewed by Walther-Rasmussen and Høiby (2006). However, with some exceptions, e.g., OXA-23, these enzymes are generally not inhibited by tazobactam, as seen for the four strains in the Carba NP II and Blue-CARBA tests and generally don't mediate resistance to aztreonam (Walther-Rasmussen and Høiby, 2006).

So far, the identity of the enzyme responsible for imipenem hydrolyzing activity in the four strains remains unclear, as none of the examined carbapenemase and AmpC-β-lactamase genes could be detected. It cannot be excluded that in addition to a carbapenem hydrolyzing enzyme other resistance mechanisms, such as reduced affinity of PBPs, porin alterations resulting in decreased membrane permeability or active efflux systems, either alone or in combination may also contribute to the observed phenotype (Walther-Rasmussen and Høiby, 2006; Queenan and Bush, 2007; Nordmann et al., 2012). Carbapenem-resistant *V. cholera*e have already been reported in other studies. NDM-1 carbapenemase was detected in a *V. cholerae* O1 El Tor Ogawa strain isolated from a 2-year old child (Mandal et al., 2012) as well as in *V. cholerae* isolated from seepage water in India (Walsh et al., 2011). Furthermore, increasing resistance to carbapenems was recently described among *V. cholerae* O1 or O139 strains isolated between 1986 and 2012 in southwest China (Gu et al., 2014).

## Concluding remarks

In this study, antimicrobial agents recommended as first choice agents for the treatment of *V. vulnificus* and *V. cholerae* non-O1/non-O139 infections such as fluoroquinolones, tetracyclines, and extended spectrum cephalosporins were found to be effective *in vitro* against both species. However, the administration of aminopenicillins, carbapenems, or aminoglycosides for treatment of *V. cholerae* non-O1/non-O139 infections, which has been reported in few studies (Daniels and Shafaie, 2000; Feghali and Adib, 2011; Lu et al., 2014) should be considered carefully, as non-susceptibility was most frequently observed against ampicillin and streptomycin and sporadically to carbapenems. For *V. vulnificus*, non-susceptibility was exclusively observed to the aminoglycosides streptomycin and kanamycin. However, gentamicin was effective against both species and could be an aminoglycoside of choice for the treatment of children and pregnant woman, as was also suggested by others (Shaw et al., 2014).

We report the detection of carbapenemase producing *V. cholerae* from different locations of the German coast line (North Sea and Baltic Sea) representing an environmental reservoir of carbapenem resistance. An entry into the sea resulting from sanitary pollution or human recreational activities cannot be excluded, but seems not likely as vibrios are indigenous bacteria of the marine environment and not intestinal commensals of humans or terrestrial animals. The strains displaying carbapenemase activity showed resistance to an unusual pattern of β-lactams. Therefore, characterization of the underlying genetic background is necessary to identify the responsible genes e.g., using whole genome sequencing as the most promising approach. Further investigations on the mobility as well as on the location of encoding genes are also needed, since location on mobile genetic elements would imply a higher risk for interspecies spread. Carbapenems are last line antimicrobial agents for treatment of multidrug-resistant Gram-negative bacteria and are of high therapeutic value (Nordmann et al., 2012). The occurrence of putative carbapenemase producing *V. cholerae* in the North and Baltic Sea is therefore of great concern and highlights the need for systematic monitoring of antimicrobial susceptibility in potentially pathogenic *Vibrio* spp. in Europe.

## Funding

This work was supported by the Federal Ministry of Education and Research (VibrioNet, BMBF grant 01KI1015A) and the Federal Institute for Risk Assessment (BfR 46-001). The German research program KLIWAS was funded by the Federal Ministry of Transport and Digital Infrastructure.

### Conflict of interest statement

The authors declare that the research was conducted in the absence of any commercial or financial relationships that could be construed as a potential conflict of interest.

## Footnotes

^1^http://www.cdc.gov/cholera/non-01-0139-infections.html

^2^http://emergency.cdc.gov/disasters/vibriovulnificus.asp

^3^http://www.kliwas.de/KLIWAS/EN/03_ResearchTasks/03_vh3/04_304/304_node.html

^4^http://www.vibrionet.de/

^5^http://www.eucast.org

## References

[B1] Andrews J. M. (2009). BSAC standardized disc susceptibility testing method (version 8). J. Antimicrob. Chemother. 64, 454–489. 10.1093/jac/dkp244 19587067

[B2] Baker-Austin C. McArthur J. V. Lindell A. H. Wright M. S. Tuckfield R. C. Gooch J.. (2009). Multi-site analysis reveals widespread antibiotic resistance in the marine pathogen *Vibrio vulnificus*. Microb. Ecol. 57, 151–159. 10.1007/s00248-008-9413-8 18642041

[B3] Baker-Austin C. Trinanes J. A. Taylor N. G. H. Hartnell R. Siitonen A. Martinez-Urtaza J. (2012). Emerging *Vibrio* risk at high latitudes in response to ocean warming. Nat. Clim. Change 3, 73–77. 10.1038/nclimate1628

[B4] Bauer A. Røervik L. M. (2007). A novel multiplex PCR for the identification of *Vibrio parahaemolyticus, Vibrio cholerae* and *Vibrio vulnificus*. Lett. Appl. Microbiol. 45, 371–375. 10.1111/j.1472-765X.2007.02195.x 17897378

[B5] Bier N. Bechlars S. Diescher S. Klein F. Hauk G. Duty O.. (2013). Genotypic diversity and virulence characteristics of clinical and environmental *Vibrio vulnificus* isolates from the baltic sea region. Appl. Environ. Microbiol. 79, 3570–3581. 10.1128/AEM.00477-13 23542621 PMC3675912

[B6] Böer S. Hauk G. Duty O. Luden K. Heinemeyer E.-A. Brennholt N. (2012). Pathogenic *Vibrio* species in German coastal waters of the North Sea and the Baltic Sea – a comparison, in Veranstaltungen International Symposium “Pathogenic Vibrio spp.in Northern European Waters” (Koblenz: German Federal Institute of Hydrology), 36–42. 10.5675/BfG_Veranst_2012.4

[B7] Ceccarelli D. Chen A. Hasan N. A. Rashed S. M. Huq A. Colwell R. R. (2015). Non-O1/non-O139 *Vibrio cholerae* carrying multiple virulence factors and *V. cholerae* O1 in the Chesapeake Bay, Maryland. Appl. Environ. Microbiol. 81, 1909–1918. 10.1128/AEM.03540-14 25556194 PMC4345386

[B8] Chen S. C. Lee Y. T. Tsai S. J. Chan K. S. Chao W. N. Wang P. H.. (2012). Antibiotic therapy for necrotizing fasciitis caused by *Vibrio vulnificus*: retrospective analysis of an 8 year period. J. Antimicrob. Chemother. 67, 488–493. 10.1093/jac/dkr476 22117030

[B9] Chen X. Naren G. W. Wu C. M. Wang Y. Dai L. Xia L. N.. (2010). Prevalence and antimicrobial resistance of *Campylobacter* isolates in broilers from China. Vet. Microbiol. 144, 133–139. 10.1016/j.vetmic.2009.12.035 20116182

[B10] CLSI (2010a). Methods for Antimicrobial Dilution and Disk Susceptibility Testing of Infrequently Isolated or Fastidious Bacteria; Approved Guideline —Second Edition M45-A2. Wayne, PA: Clinical and Laboratory Standards Institute, CLSI.

[B11] CLSI (2010b). Performance Standards for Antimicrobial Susceptibility Testing; Twentieth Informational Supplement M100-S20. Wayne, PA: Clinical and Laboratory Standards Institute, CLSI.

[B12] CLSI (2012a). Methods for Dilution Antimicrobial Susceptibility Tests for Bacteria That Grow Aerobically; Approved Standard—Ninth Edition M7-A9. Wayne, PA: Clinical and Laboratory Standards Institute, CLSI.

[B13] CLSI (2012b). Performance Standards for Antimicrobial Disk Susceptibility Tests; Approved Standard—Eleventh Edition M2-A11. Wayne, PA: Clinical and Laboratory Standards Institute, CLSI.

[B14] CLSI (2013). Performance Standards for Antimicrobial Disk and Dilution Susceptibility Tests for Bacteria Isolated From Animals; Second Informational Supplement VET01-S2. Wayne, PA: Clinical and Laboratory Standards Institute, CLSI.

[B15] CLSI (2015). Performance Standards for Antimicrobial Susceptibility Testing; Twenty-fifth Informational Supplement M100-S25. Wayne, PA: Clinical and Laboratory Standards Institute, CLSI.

[B16] Daniels N. A. Shafaie A. (2000). A review of pathogenic *Vibrio* infections for clinicians. Infect. Med. 17, 665–685.

[B17] Dortet L. Bréchard L. Cuzon G. Poirel L. Nordmann P. (2014). Strategy for rapid detection of carbapenemase-producing *Enterobacteriaceae*. Antimicrob. Agents Chemother. 58, 2441–2445. 10.1128/AAC.01239-13 24468779 PMC4023735

[B18] Dortet L. Poirel L. Nordmann P. (2012). Rapid identification of carbapenemase types in *Enterobacteriaceae* and *Pseudomonas* spp. By using a biochemical test. Antimicrob. Agents. Chemother. 56, 6437–6440. 10.1128/AAC.01395-12 23070158 PMC3497194

[B19] EUCAST (2015). The European Committee on Antimicrobial Susceptibility Testing. Breakpoint Tables for Interpretation of MICs and Zone Diameters. Version 5.0. Available online at: http://www.eucast.org.

[B20] Farmer J. J. III Janda J. M. (2004). Family I. *Vibrionaceae*, in Bergey's Manual of Systematic Bacteriology, 2nd Edn., ed Garrity G. M. (New York, NY: Springer), 491–546.

[B21] Feghali R. Adib S. M. (2011). Two cases of *Vibrio cholerae* non-O1/non-O139 septicaemia with favourable outcome in Lebanon. East. Mediterr. Health J. 17, 722–724. 21977578

[B22] Foster T. J. (1983). Plasmid-determined resistance to antimicrobial drugs and toxic metal ions in bacteria. Microbiol. Rev. 47, 361–409. 6355806 10.1128/mr.47.3.361-409.1983 PMC281581

[B23] Gu W. Yin J. Yang J. Li C. Chen Y. Yin J.. (2014). Characterization of *Vibrio cholerae* from 1986 to 2012 in Yunnan Province, southwest China bordering Myanmar. Infect. Genet. Evol. 21, 1–7. 10.1016/j.meegid.2013.10.015 24177595

[B24] Han F. Walker R. D. Janes M. E. Prinyawiwatkul W. Ge B. (2007). Antimicrobial susceptibilities of *Vibrio parahaemolyticus* and *Vibrio vulnificus* isolates from Louisiana Gulf and retail raw oysters. Appl. Environ. Microbiol. 73, 7096–7098. 10.1128/AEM.01116-07 17827331 PMC2074966

[B25] Hochhut B. Lotfi Y. Mazel D. Faruque S. M. Woodgate R. Waldor M. K. (2001). Molecular analysis of antibiotic resistance gene clusters in *Vibrio cholerae* O139 and O1 SXT constins. Antimicrob. Agents Chemother. 45, 2991–3000. 10.1128/AAC.45.11.2991-3000.2001 11600347 PMC90773

[B26] Huehn S. Eichhorn C. Urmersbach S. Breidenbach J. Bechlars S. Bier N.. (2014). Pathogenic vibrios in environmental, seafood and clinical sources in Germany. Int. J. Med. Microbiol. 304, 843–850. 10.1016/j.ijmm.2014.07.010 25129553

[B27] Huhulescu S. Indra A. Feierl G. Stoeger A. Ruppitsch W. Sarkar B.. (2007). Occurrence of *Vibrio cholerae* serogroups other than O1 and O139 in Austria. Wien. Klin. Wochenschr. 119, 235–241. 10.1007/s00508-006-0747-2 17492351

[B28] Kaper J. B. Morris J. G. Jr. Levine M. M. (1995). Cholera. Clin. Microbiol. Rev. 8, 48–86. 7704895 10.1128/cmr.8.1.48 PMC172849

[B29] Kumar P. A. Patterson J. Karpagam P. (2009). Multiple antibiotic resistance profiles of *Vibrio cholerae* non-O1 and non-O139. Jpn. J. Infect. Dis. 62, 230–232. 19468189

[B30] Letchumanan V. Chan K. G. Lee L. H. (2014). *Vibrio parahaemolyticus*: a review on the pathogenesis, prevalence, and advance molecular identification techniques. Front. Microbiol. 5:705. 10.3389/fmicb.2014.00705 25566219 PMC4263241

[B31] Lu B. Zhou H. Li D. Li F. Zhu F. Cui Y.. (2014). The first case of bacteraemia due to non-O1/non-O139 *Vibrio cholerae* in a type 2 diabetes mellitus patient in mainland China. Int. J. Infect. Dis. 25, 116–118. 10.1016/j.ijid.2014.04.015 24905769

[B32] Luo Y. Ye J. Jin D. Ding G. Zhang Z. Mei L.. (2013). Molecular analysis of non-O1/non-O139 *Vibrio cholerae* isolated from hospitalised patients in China. BMC Microbiol. 13:52. 10.1186/1471-2180-13-52 23497008 PMC3605376

[B33] Lutz C. Erken M. Noorian P. Sun S. McDougald D. (2013). Environmental reservoirs and mechanisms of persistence of *Vibrio cholerae*. Front. Microbiol. 4:375. 10.3389/fmicb.2013.00375 24379807 PMC3863721

[B34] Mandal J. Sangeetha V. Ganesan V. Parveen M. Preethi V. Harish B. N.. (2012). Third-generation cephalosporin-resistant *Vibrio cholerae*, India. Emerg. Infect. Dis. 18, 1326–1328. 10.3201/eid1808.111686 22840562 PMC3414027

[B35] Mansergh S. Zehr J. P. (2014). *Vibrio* diversity and dynamics in the Monterey Bay upwelling region. Front. Microbiol. 5:48. 10.3389/fmicb.2014.00048 24575086 PMC3921578

[B36] Massad G. Oliver J. D. (1987). New selective and differential medium for *Vibrio cholerae* and *Vibrio vulnificus*. Appl. Environ. Microbiol. 53, 2262–2264. 3674873 10.1128/aem.53.9.2262-2264.1987 PMC204093

[B37] National Food Institute T. U. O. D. (2013). DANMAP 2013. Use of Antimicrobial Agents and Occurrence of Antimicrobial Resistance in Bacteria from Food Animals, Food and Humans in Denmark.

[B38] Nordmann P. Dortet L. Poirel L. (2012). Carbapenem resistance in *Enterobacteriaceae*: here is the storm! Trends Mol. Med. 18, 263–272. 10.1016/j.molmed.2012.03.003 22480775

[B39] Oliver J. D. (2006). “Vibrio vulnificus,” in *The Biology of Vibrios*, eds Thompson F. L. Austin B. Swings J. 349–366. 10.1128/9781555815714.ch25

[B40] Petsaris O. Nousbaum J. B. Quilici M. L. Le Coadou G. Payan C. Abalain M. L. (2010). Non-O1, non-O139 *Vibrio cholerae* bacteraemia in a cirrhotic patient. J. Med. Microbiol. 59, 1260–1262. 10.1099/jmm.0.021014-0 20616193

[B41] Pires J. Novais A. Peixe L. (2013). Blue-carba, an easy biochemical test for detection of diverse carbapenemase producers directly from bacterial cultures. J. Clin. Microbiol. 51, 4281–4283. 10.1128/JCM.01634-13 24108615 PMC3838089

[B42] Queenan A. M. Bush K. (2007). Carbapenemases: the versatile beta-lactamases. Clin. Microbiol. Rev. 20, 440–458. 10.1128/CMR.00001-07 17630334 PMC1932750

[B43] Sá L. L. Fonseca E. L. Pellegrini M. Freitas F. Loureiro E. C. Vicente A. C. (2010). Occurrence and composition of class 1 and class 2 integrons in clinical and environmental O1 and non-O1/non-O139 *Vibrio cholerae* strains from the Brazilian Amazon. Mem. Inst. Oswaldo Cruz 105, 229–232. 10.1590/S0074-02762010000200021 20428687

[B44] Schirmeister F. Dieckmann R. Bechlars S. Bier N. Faruque S. M. Strauch E. (2014). Genetic and phenotypic analysis of *Vibrio cholerae* non-O1, non-O139 isolated from German and Austrian patients. Eur. J. Clin. Microbiol. Infect. Dis. 33, 767–778. 10.1007/s10096-013-2011-9 24213848 PMC3996285

[B45] Shapiro R. L. Altekruse S. Hutwagner L. Bishop R. Hammond R. Wilson S.. (1998). The role of Gulf Coast oysters harvested in warmer months in *Vibrio vulnificus* infections in the United States, 1988-1996. Vibrio Working Group. J. Infect. Dis. 178, 752–759. 10.1086/515367 9728544

[B46] Shaw K. J. Rather P. N. Hare R. S. Miller G. H. (1993). Molecular genetics of aminoglycoside resistance genes and familial relationships of the aminoglycoside-modifying enzymes. Microbiol. Rev. 57, 138–163. 8385262 10.1128/mr.57.1.138-163.1993 PMC372903

[B47] Shaw K. S. Rosenberg Goldstein R. E. He X. Jacobs J. M. Crump B. C. Sapkota A. R. (2014). Antimicrobial susceptibility of *Vibrio vulnificus* and *Vibrio parahaemolyticus* recovered from recreational and commercial areas of Chesapeake Bay and Maryland Coastal Bays. PloS ONE 9:e89616. 10.1371/journal.pone.0089616 24586914 PMC3934932

[B48] Thompson J. R. Polz M. F. (2006). Dynamics of vibrio populations and their role in environmental nutrient cycling, in The Biology of Vibrios, eds Thompson F. L. Austin B. Swings J. (Washington, DC: ASM Press), 190–203. 10.1128/9781555815714.ch13

[B49] Tobin-D'Angelo M. Smith A. R. Bulens S. N. Thomas S. Hodel M. Izumiya H.. (2008). Severe diarrhea caused by cholera toxin-producing *Vibrio cholerae* serogroup O75 infections acquired in the southeastern United States. Clin. Infect. Dis. 47, 1035–1040. 10.1086/591973 18781876

[B50] Tsai Y. K. Liou C. H. Lin J. C. Ma L. Fung C. P. Chang F. Y.. (2014). A suitable streptomycin-resistant mutant for constructing unmarked in-frame gene deletions using rpsL as a counter-selection marker. PLoS ONE 9:e109258. 10.1371/journal.pone.0109258 25268958 PMC4182516

[B51] Walsh T. R. Weeks J. Livermore D. M. Toleman M. A. (2011). Dissemination of NDM-1 positive bacteria in the New Delhi environment and its implications for human health: an environmental point prevalence study. Lancet Infect. Dis. 11, 355–362. 10.1016/S1473-3099(11)70059-7 21478057

[B52] Walther-Rasmussen J. Høiby N. (2006). OXA-type carbapenemases. J. Antimicrob. Chemother. 57, 373–383. 10.1093/jac/dki482 16446375

[B53] Yu L. Zhou Y. Wang R. Lou J. Zhang L. Li J.. (2012). Multiple antibiotic resistance of *Vibrio cholerae* serogroup O139 in China from 1993 to 2009. PLoS ONE 7:e38633. 10.1371/journal.pone.0038633 22701685 PMC3372494

