# Supplementary material for: Survey on antimicrobial resistance patterns in Vibrio vulnificus and Vibrio cholerae non-O1/non-O139 in Germany reveals carbapenemase-producing Vibrio cholerae in coastal waters
Source: Front Microbiol. 2015 Oct 28;6:1179. doi: 10.3389/fmicb.2015.01179 (PMC4623411; doi:10.3389/fmicb.2015.01179)
Supplement: Supplementary file 1 [file Table1.PDF]

Supplementary Material

Survey on antimicrobial resistance patterns in *Vibrio vulnificus* and *Vibrio cholerae* non-O1/non-O139 in Germany reveals carbapenemase-producing *Vibrio cholerae* in coastal waters

Nadja Bier, Keike Schwartz, Beatriz Guerra, and Eckhard Strauch*

*Correspondence: Eckhard Strauch; **eckhard.strauch@bfr.bund.de**

1 Supplementary Tables

Supplementary Table S1. Results of broth microdilution and disk diffusion assays of all *V. vulnificus* isolates[a]

	Strain source code[b]	Year	Minimal inhibitory concentration (MIC) [mg/L]													Inhibition zone diameter [mm]						Resistance Profile[c]
			AMP R ≥32	CHL R ≥32	CIP R ≥4	CST R >2	FFN R ≥16	CTX R ≥4	GEN R ≥16	KAN R ≥64	NAL R ≥32	STR R ≥64	CAZ R ≥16	TET R ≥16	TMP R ≥16	AMC R ≤13	SXT R ≤10	FEP R ≤18	LVX R ≤13	MEM R ≤19	IPM R ≤19	
**Clinical**																						
VN-0094	C-D-ext	1994	1	≤2	0.03	**>4**	≤2	≤0.06	1	≤4	≤4	16	≤0.25	≤1	2	27	30	28	34	32	32	susceptible
VN-0095	C-D-ext	1994	1	≤2	0.015	**>4**	≤2	≤0.06	1	8	≤4	16	≤0.25	≤1	1	28	30	32	36	36	36	susceptible
VN-0096	C-D-ext	1994	1	≤2	0.015	**>4**	≤2	≤0.06	0.5	≤4	≤4	4	≤0.25	≤1	≤0.5	28	35	30	32	40	34	susceptible
VN-0097	C-D-ext	1994	2	≤2	0.015	**>4**	≤2	0.12	4	16	≤4	**32**	0.5	≤1	≤0.5	30	30	22	32	24	28	(STR)
VN-0098	C-D-ext	1994	2	≤2	0.015	**>4**	≤2	0.12	4	16	≤4	**64**	0.5	≤1	1	22	34	26	32	32	31	STR
VN-0125	C-D-ext	1994	2	≤2	0.03	**>4**	≤2	0.12	4	**32**	≤4	**64**	0.5	≤1	1	24	30	26	32	32	34	STR, (KAN)
VN-0126	C-D-ext	1994	1	≤2	0.015	**>4**	≤2	≤0.06	1	≤4	≤4	16	≤0.25	≤1	≤0.5	27	34	32	36	34	34	susceptible
VN-0127	C-D-ext	1994	1	≤2	0.015	**>4**	≤2	≤0.06	1	8	≤4	**32**	0.5	≤1	1	27	32	30	38	38	32	(STR)
VN-0128	C-D-ext	1994	1	≤2	0.03	**>4**	≤2	≤0.06	4	16	≤4	**32**	≤0.25	≤1	2	26	32	30	40	34	32	(STR)
VN-0129	C-D-ext	1994	2	≤2	0.03	**>4**	≤2	≤0.06	4	16	≤4	**64**	≤0.25	≤1	4	34	30	34	40	34	32	STR
VN-0130	C-D-ext	1994	1	≤2	0.015	**>4**	≤2	≤0.06	2	16	≤4	**32**	≤0.25	≤1	≤0.5	34	34	30	36	40	36	(STR)
VN-0131	C-D-ext	1994	1	≤2	0.03	**>4**	≤2	≤0.06	4	8	≤4	16	≤0.25	≤1	1	27	31	28	34	36	32	susceptible
VN-0132	C-D-ext	1994	2	≤2	0.015	**>4**	≤2	≤0.06	1	8	≤4	**32**	≤0.25	≤1	1	28	33	32	40	36	34	(STR)
VN-0133	C-D-ext	1994	1	≤2	0.015	**>4**	≤2	≤0.06	2	8	≤4	**32**	≤0.25	≤1	1	27	31	30	38	36	32	(STR)
VN-0010	C-G-ext	1994	2	≤2	0.015	**>4**	≤2	≤0.06	1	8	≤4	16	≤0.25	≤1	1	24	28	26	26	32	28	susceptible
VN-0108	C-G-ext	2010	2	≤2	≤0.008	**>4**	≤2	≤0.06	2	8	≤4	16	≤0.25	≤1	≤0.5	29	32	30	38	38	34	susceptible
VN-0112	C-G-ext	2010	1	≤2	0.06	**>4**	≤2	≤0.06	4	8	≤4	**32**	≤0.25	≤1	1	26	31	30	38	36	32	(STR)
VN-0288	C-G-ext	2010	1	≤2	0.015	**>4**	≤2	≤0.06	1	8	≤4	16	≤0.25	≤1	1	38	38	38	40	44	40	susceptible
VN-0092	C-G-ext	2011	1	≤2	0.015	**>4**	≤2	≤0.06	1	16	≤4	**32**	≤0.25	≤1	≤0.5	26	31	26	36	37	34	(STR)
**Environmental Baltic Sea**																						
VN-3979	E-BS-sd	2010	1	≤2	0.015	**>4**	≤2	≤0.06	4	8	≤4	**32**	≤0.25	≤1	1	30	30	30	34	36	36	(STR)
VN-3904	E-BS-sd	2011	1	≤2	0.015	**>4**	≤2	≤0.06	2	8	≤4	**32**	≤0.25	≤1	≤0.5	26	30	26	30	32	32	(STR)
VN-3905	E-BS-sd	2011	1	≤2	0.015	**>4**	≤2	≤0.06	1	≤4	≤4	16	≤0.25	≤1	1	26	29	28	32	36	30	susceptible
VN-3906	E-BS-sd	2011	1	≤2	0.03	**>4**	≤2	≤0.06	2	8	≤4	16	0.5	≤1	1	26	30	28	36	36	34	susceptible
VN-3909	E-BS-sd	2011	2	≤2	0.015	**>4**	≤2	≤0.06	2	≤4	≤4	16	≤0.25	≤1	≤0.5	30	32	32	34	36	36	susceptible
VN-3910	E-BS-sd	2011	1	≤2	≤0.008	**>4**	≤2	≤0.06	1	≤4	≤4	16	≤0.25	≤1	≤0.5	28	32	34	34	34	34	susceptible
VN-3912	E-BS-sd	2011	1	≤2	0.015	**>4**	≤2	≤0.06	0.5	≤4	≤4	8	≤0.25	≤1	1	34	34	34	50	50	44	susceptible
VN-3914	E-BS-sd	2011	1	≤2	0.015	**>4**	≤2	≤0.06	1	≤4	≤4	**32**	≤0.25	≤1	≤0.5	34	34	34	36	40	42	(STR)
VN-3915	E-BS-sd	2011	1	≤2	≤0.008	**>4**	≤2	≤0.06	1	8	≤4	16	≤0.25	≤1	1	30	32	28	34	34	34	susceptible
VN-3919	E-BS-sd	2011	1	≤2	0.015	**>4**	≤2	≤0.06	2	8	≤4	16	≤0.25	≤1	≤0.5	27	32	26	32	34	34	susceptible
VN-3921	E-BS-sd	2011	1	≤2	0.015	**>4**	≤2	≤0.06	1	≤4	≤4	16	≤0.25	≤1	≤0.5	29	30	30	30	40	36	susceptible
VN-3924	E-BS-sd	2011	2	≤2	0.03	**>4**	≤2	0.12	1	≤4	≤4	16	0.5	≤1	1	25	30	29	32	30	30	susceptible
VN-3925	E-BS-sd	2011	2	≤2	0.03	**>4**	≤2	≤0.06	2	8	≤4	16	≤0.25	≤1	≤0.5	25	29	27	34	32	32	susceptible
VN-3926	E-BS-sd	2011	1	≤2	0.015	**>4**	≤2	≤0.06	1	8	≤4	16	≤0.25	≤1	1	26	29	30	32	36	32	susceptible
VN-3927	E-BS-sd	2011	1	≤2	0.015	**>4**	≤2	≤0.06	2	16	≤4	**32**	≤0.25	≤1	≤0.5	30	32	30	36	36	34	(STR)
VN-3929	E-BS-sd	2011	2	≤2	0.015	**>4**	≤2	≤0.06	1	≤4	≤4	16	≤0.25	≤1	1	26	30	30	34	26	30	susceptible
VN-3931	E-BS-sd	2011	1	≤2	0.015	**>4**	≤2	≤0.06	1	≤4	≤4	**32**	≤0.25	≤1	1	26	30	28	32	34	34	(STR)

Table continued

	Strain source code[b]	Year	Minimal inhibitory concentration (MIC) [mg/L]													Inhibition zone diameter [mm]						Resistance Profile[c]
			AMP R ≥32	CHL R ≥32	CIP R ≥4	CST R >2	FFN R ≥16	CTX R ≥4	GEN R ≥16	KAN R ≥64	NAL R ≥32	STR R ≥64	CAZ R ≥16	TET R ≥16	TMP R ≥16	AMC R ≤13	SXT R ≤10	FEP R ≤18	LVX R ≤13	MEM R ≤19	IPM R ≤19	
VN-3932	E-BS-sd	2011	1	≤2	0.015	**>4**	≤2	≤0.06	2	8	≤4	**32**	≤0.25	≤1	2	26	28	26	32	32	32	(STR)
VN-3934	E-BS-sd	2011	1	≤2	0.03	**>4**	≤2	≤0.06	2	8	≤4	**32**	≤0.25	≤1	≤0.5	26	29	30	36	36	34	(STR)
VN-3935	E-BS-sd	2011	1	≤2	0.015	**>4**	≤2	≤0.06	1	≤4	≤4	16	≤0.25	≤1	≤0.5	27	32	30	38	34	34	susceptible
VN-3937	E-BS-sd	2011	1	≤2	0.015	**>4**	≤2	≤0.06	2	8	≤4	**32**	0.5	≤1	1	25	30	32	28	32	30	(STR)
VN-3946	E-BS-sd	2011	1	≤2	0.015	**>4**	≤2	≤0.06	2	8	≤4	**32**	≤0.25	≤1	≤0.5	28	32	30	34	40	34	(STR)
VN-3947	E-BS-sd	2011	1	≤2	0.015	**>4**	≤2	≤0.06	1	≤4	≤4	16	≤0.25	≤1	≤0.5	28	30	29	32	38	34	susceptible
VN-3948	E-BS-sd	2011	1	≤2	0.03	**>4**	≤2	≤0.06	2	8	≤4	**32**	≤0.25	≤1	≤0.5	25	28	28	30	34	32	(STR)
VN-0227	E-BS-sw	2004	1	≤2	0.015	**>4**	≤2	≤0.06	2	8	≤4	**32**	≤0.25	≤1	1	34	40	50	50	50	50	(STR)
VN-0235	E-BS-sw	2006	1	≤2	0.015	**>4**	≤2	≤0.06	1	≤4	≤4	16	≤0.25	≤1	≤0.5	26	32	26	32	36	32	susceptible
VN-0239	E-BS-sw	2006	2	≤2	0.015	**>4**	≤2	≤0.06	2	8	≤4	**32**	0.5	≤1	≤0.5	25	32	30	34	34	30	(STR)
VN-0243	E-BS-sw	2006	1	≤2	≤0.008	**>4**	≤2	≤0.06	0.5	≤4	≤4	8	≤0.25	≤1	1	28	30	30	36	36	30	susceptible
VN-0251	E-BS-sw	2006	1	≤2	0.015	**>4**	≤2	≤0.06	2	8	≤4	**32**	≤0.25	≤1	1	25	31	26	30	32	34	(STR)
VN-0260	E-BS-sw	2007	≤0.5	≤2	0.015	**>4**	≤2	≤0.06	1	≤4	≤4	16	≤0.25	≤1	1	36	34	34	40	44	44	susceptible
VN-0264	E-BS-sw	2007	1	≤2	0.015	**>4**	≤2	≤0.06	1	≤4	≤4	8	≤0.25	≤1	≤0.5	33	33	30	40	40	40	susceptible
VN-0266	E-BS-sw	2007	1	≤2	≤0.008	**>4**	≤2	≤0.06	2	8	≤4	**32**	≤0.25	≤1	1	25	28	25	32	33	34	(STR)
VN-0270	E-BS-sw	2008	1	≤2	0.015	**>4**	≤2	≤0.06	2	8	≤4	16	≤0.25	≤1	≤0.5	30	32	28	32	36	34	susceptible
VN-0274	E-BS-sw	2008	1	≤2	≤0.008	**>4**	≤2	≤0.06	2	8	≤4	16	≤0.25	≤1	1	28	32	28	32	36	34	susceptible
VN-0275	E-BS-sw	2008	1	≤2	0.015	**>4**	≤2	≤0.06	1	8	≤4	16	≤0.25	≤1	≤0.5	28	34	28	36	36	34	susceptible
VN-0276	E-BS-sw	2008	2	≤2	0.03	**>4**	≤2	≤0.06	2	8	≤4	16	≤0.25	≤1	2	28	31	28	34	36	36	susceptible
VN-0277	E-BS-sw	2008	1	≤2	0.015	**>4**	≤2	≤0.06	2	8	≤4	16	≤0.25	≤1	1	28	31	30	31	38	34	susceptible
VN-0100	E-BS-sw	2010	1	≤2	0.015	**>4**	≤2	0.12	2	8	≤4	**64**	≤0.25	≤1	1	28	30	28	34	38	34	STR
VN-0101	E-BS-sw	2010	2	≤2	≤0.008	**>4**	≤2	≤0.06	1	8	≤4	16	≤0.25	≤1	1	26	28	28	34	34	33	susceptible
VN-0102	E-BS-sw	2010	1	≤2	0.015	**>4**	≤2	≤0.06	1	8	≤4	16	≤0.25	≤1	≤0.5	26	29	22	30	32	32	susceptible
VN-0103	E-BS-sw	2010	1	≤2	0.015	**>4**	≤2	≤0.06	2	8	≤4	16	≤0.25	≤1	≤0.5	26	30	28	32	34	30	susceptible
VN-0104	E-BS-sw	2010	1	≤2	0.015	**>4**	≤2	≤0.06	2	8	≤4	16	≤0.25	≤1	1	29	29	28	32	34	34	susceptible
VN-0105	E-BS-sw	2010	2	≤2	0.015	**>4**	≤2	≤0.06	2	16	≤4	16	0.5	2	1	22	26	26	34	32	30	susceptible
VN-3959	E-BS-sw	2010	1	≤2	0.015	**>4**	≤2	≤0.06	2	8	≤4	**32**	≤0.25	≤1	1	28	30	30	30	34	34	(STR)
VN-3960	E-BS-sw	2010	1	≤2	0.03	**>4**	≤2	≤0.06	1	8	≤4	**32**	0.5	≤1	1	30	31	28	32	38	34	(STR)
VN-3961	E-BS-sw	2010	1	≤2	0.015	**>4**	≤2	≤0.06	1	8	≤4	16	≤0.25	≤1	≤0.5	25	31	26	30	36	32	susceptible
VN-3962	E-BS-sw	2010	1	≤2	≤0.008	**>4**	≤2	≤0.06	1	≤4	≤4	8	≤0.25	≤1	≤0.5	30	34	50	50	40	30	susceptible
VN-3964	E-BS-sw	2010	1	≤2	0.015	**>4**	≤2	≤0.06	2	16	≤4	**32**	≤0.25	≤1	1	30	30	30	34	36	36	(STR)
VN-3965	E-BS-sw	2010	1	≤2	≤0.008	**>4**	≤2	≤0.06	4	8	≤4	**32**	≤0.25	≤1	≤0.5	28	34	36	34	30	30	(STR)
VN-3966	E-BS-sw	2010	1	≤2	0.015	**>4**	≤2	≤0.06	2	8	≤4	**32**	≤0.25	≤1	1	32	34	34	38	40	38	(STR)
VN-3968	E-BS-sw	2010	1	≤2	0.015	**>4**	≤2	≤0.06	1	≤4	≤4	**32**	≤0.25	≤1	1	30	31	32	32	36	34	(STR)
VN-3969	E-BS-sw	2010	2	≤2	0.015	**>4**	≤2	≤0.06	2	16	≤4	16	≤0.25	≤1	1	26	30	28	36	36	36	susceptible
VN-3970	E-BS-sw	2010	2	≤2	≤0.008	**>4**	≤2	≤0.06	2	8	≤4	**32**	≤0.25	≤1	1	24	31	28	34	34	30	(STR)
VN-3971	E-BS-sw	2010	2	≤2	0.03	**>4**	≤2	≤0.06	1	≤4	≤4	16	≤0.25	≤1	1	30	33	30	32	44	38	susceptible
VN-3972	E-BS-sw	2010	1	≤2	≤0.008	**>4**	≤2	≤0.06	2	8	≤4	16	≤0.25	≤1	1	28	32	26	38	36	34	susceptible
VN-3973	E-BS-sw	2010	1	≤2	0.015	**>4**	≤2	≤0.06	2	≤4	≤4	16	≤0.25	≤1	1	28	32	32	34	38	36	susceptible
VN-3974	E-BS-sw	2010	1	≤2	0.015	**>4**	≤2	≤0.06	2	8	≤4	**32**	≤0.25	≤1	1	28	28	26	30	34	40	(STR)

Table continued																						
	Strain source code[b]	Year	Minimal inhibitory concentration (MIC) [mg/L]													Inhibition zone diameter [mm]						Resistance Profile[c]
			AMP R ≥32	CHL R ≥32	CIP R ≥4	CST R >2	FFN R ≥16	CTX R ≥4	GEN R ≥16	KAN R ≥64	NAL R ≥32	STR R ≥64	CAZ R ≥16	TET R ≥16	TMP R ≥16	AMC R ≤13	SXT R ≤10	FEP R ≤18	LVX R ≤13	MEM R ≤19	IPM R ≤19	
VN-3975	E-BS-sw	2010	1	≤2	≤0.008	**>4**	≤2	≤0.06	1	≤4	≤4	16	≤0.25	≤1	≤0.5	28	30	26	32	34	35	susceptible
VN-3976	E-BS-sw	2010	1	≤2	≤0.008	**4**	≤2	≤0.06	1	≤4	≤4	16	≤0.25	≤1	1	24	29	26	32	34	32	susceptible
VN-3977	E-BS-sw	2010	1	≤2	≤0.008	**>4**	≤2	≤0.06	1	8	≤4	16	≤0.25	≤1	1	26	29	27	32	34	32	susceptible
VN-3978	E-BS-sw	2010	1	≤2	0.03	**>4**	≤2	≤0.06	1	8	≤4	16	≤0.25	≤1	1	26	30	28	32	36	32	susceptible
VN-3980	E-BS-sw	2010	1	≤2	≤0.008	**>4**	≤2	≤0.06	1	8	≤4	16	≤0.25	≤1	≤0.5	28	31	30	32	32	34	susceptible
VN-3982	E-BS-sw	2010	1	≤2	0.015	**>4**	≤2	≤0.06	2	8	≤4	16	≤0.25	≤1	≤0.5	28	33	30	34	36	34	susceptible
VN-0279	E-BS-sw	2011	2	≤2	0.03	**>4**	≤2	≤0.06	2	8	≤4	**32**	≤0.25	≤1	1	27	30	30	30	30	30	(STR)
VN-0280	E-BS-sw	2011	1	≤2	0.015	**>4**	≤2	≤0.06	2	8	≤4	16	≤0.25	≤1	1	28	30	28	32	34	34	susceptible
VN-2961	E-BS-sw	2011	≤0.5	≤2	0.03	**>4**	≤2	≤0.06	1	≤4	≤4	16	≤0.25	≤1	≤0.5	30	32	30	32	32	30	susceptible
VN-2969	E-BS-sw	2011	≤0.5	≤2	0.03	**>4**	≤2	≤0.06	1	8	≤4	16	≤0.25	≤1	≤0.5	30	32	30	34	40	36	susceptible
VN-3922	E-BS-sw	2011	1	≤2	0.03	**>4**	≤2	≤0.06	4	16	≤4	**32**	≤0.25	≤1	1	27	29	30	30	33	30	(STR)
VN-3928	E-BS-sw	2011	1	≤2	0.015	**>4**	≤2	≤0.06	1	8	≤4	16	≤0.25	≤1	1	24	27	28	30	30	30	susceptible
VN-3981	E-BS-sw	2011	1	≤2	0.015	**>4**	≤2	≤0.06	2	8	≤4	**32**	≤0.25	≤1	1	26	30	28	30	32	31	(STR)
**Environmental North Sea**																						
VN-10119	E-NS-bm	2012	1	≤2	≤0.008	**>4**	≤2	≤0.06	2	8	≤4	16	≤0.25	≤1	1	28	31	28	32	36	34	susceptible
VN-10121	E-NS-bm	2012	1	≤2	0.015	**>4**	≤2	≤0.06	2	16	≤4	**32**	≤0.25	≤1	1	27	30	30	36	38	34	(STR)
VN-3363	E-NS-sd	2010	1	≤2	0.015	**>4**	≤2	≤0.06	2	8	≤4	16	≤0.25	≤1	1	27	30	30	38	40	34	susceptible
VN-3364	E-NS-sd	2010	1	≤2	0.015	**>4**	≤2	≤0.06	2	8	≤4	16	≤0.25	≤1	1	26	30	28	31	34	32	susceptible
VN-3366	E-NS-sd	2010	≤0.5	≤2	0.015	**>4**	≤2	≤0.06	1	≤4	≤4	16	≤0.25	≤1	1	40	40	42	40	48	45	susceptible
VN-3373	E-NS-sd	2010	1	≤2	0.015	**>4**	≤2	≤0.06	2	8	≤4	**32**	≤0.25	≤1	1	24	30	30	30	32	30	(STR)
VN-3374	E-NS-sd	2010	1	≤2	0.015	**>4**	≤2	≤0.06	4	8	≤4	**32**	≤0.25	≤1	1	25	30	26	30	34	30	(STR)
VN-3394	E-NS-sd	2010	1	≤2	0.03	**>4**	≤2	≤0.06	2	8	≤4	**32**	≤0.25	≤1	1	26	31	31	34	34	34	(STR)
VN-3411	E-NS-sd	2010	1	≤2	0.03	**>4**	≤2	≤0.06	4	16	≤4	**32**	≤0.25	≤1	≤0.5	29	34	28	34	40	36	(STR)
VN-3418	E-NS-sd	2010	1	≤2	0.015	**>4**	≤2	≤0.06	2	8	≤4	**32**	≤0.25	≤1	1	29	33	30	36	34	34	(STR)
VN-3426	E-NS-sd	2010	1	≤2	0.03	**>4**	≤2	≤0.06	1	≤4	≤4	16	≤0.25	≤1	1	22	32	27	32	34	30	susceptible
VN-3442	E-NS-sd	2010	2	≤2	0.03	**>4**	≤2	≤0.06	2	8	≤4	**32**	≤0.25	≤1	≤0.5	26	32	28	32	34	32	(STR)
VN-3443	E-NS-sd	2010	1	≤2	0.06	**>4**	≤2	≤0.06	2	16	≤4	16	≤0.25	≤1	≤0.5	28	33	29	32	36	32	susceptible
VN-3444	E-NS-sd	2010	1	≤2	0.03	**>4**	≤2	≤0.06	2	8	≤4	16	≤0.25	≤1	≤0.5	26	30	29	32	36	32	susceptible
VN-3446	E-NS-sd	2010	1	≤2	0.03	**>4**	≤2	≤0.06	2	8	≤4	16	≤0.25	≤1	1	28	30	28	30	36	32	susceptible
VN-3454	E-NS-sd	2010	1	≤2	≤0.008	**>4**	≤2	≤0.06	1	8	≤4	16	≤0.25	≤1	1	30	30	32	40	38	33	susceptible
VN-3457	E-NS-sd	2010	≤0.5	≤2	0.015	**>4**	≤2	≤0.06	1	≤4	≤4	16	≤0.25	≤1	≤0.5	32	30	30	32	38	36	susceptible
VN-3465	E-NS-sd	2010	1	≤2	0.015	**>4**	≤2	≤0.06	1	≤4	≤4	16	≤0.25	≤1	1	30	34	32	36	40	36	susceptible
VN-3467	E-NS-sd	2010	1	≤2	0.015	**>4**	≤2	≤0.06	2	8	8	16	≤0.25	≤1	1	28	32	32	38	40	36	susceptible
VN-3478	E-NS-sd	2010	1	≤2	0.015	**>4**	≤2	≤0.06	2	8	≤4	16	≤0.25	≤1	1	29	32	32	34	36	34	susceptible
VN-3479	E-NS-sd	2010	1	≤2	≤0.008	**>4**	≤2	≤0.06	2	≤4	≤4	16	≤0.25	≤1	1	32	32	36	36	40	34	susceptible
VN-3498	E-NS-sd	2010	1	≤2	0.015	**>4**	≤2	≤0.06	2	8	≤4	**32**	≤0.25	≤1	≤0.5	29	32	30	34	36	36	(STR)
VN-3500	E-NS-sd	2011	1	≤2	0.015	**>4**	≤2	≤0.06	2	8	≤4	16	≤0.25	≤1	≤0.5	28	32	30	38	34	34	susceptible
VN-3367	E-NS-sw	2010	2	≤2	0.015	**>4**	≤2	≤0.06	2	8	≤4	**32**	≤0.25	≤1	1	28	30	30	34	32	30	(STR)
VN-3368	E-NS-sw	2010	≤0.5	≤2	0.015	**>4**	≤2	≤0.06	0.5	≤4	≤4	8	≤0.25	≤1	1	50	50	50	50	50	50	susceptible
VN-3369	E-NS-sw	2010	1	≤2	0.03	**>4**	≤2	0.12	2	16	≤4	**32**	≤0.25	≤1	1	25	30	30	30	34	30	(STR)

Table continued

	Strain source code[b]	Year	Minimal inhibitory concentration (MIC) [mg/L]													Inhibition zone diameter [mm]						Resistance Profile[c]
			AMP R ≥32	CHL R ≥32	CIP R ≥4	CST R >2	FFN R ≥16	CTX R ≥4	GEN R ≥16	KAN R ≥64	NAL R ≥32	STR R ≥64	CAZ R ≥16	TET R ≥16	TMP R ≥16	AMC R ≤13	SXT R ≤10	FEP R ≤18	LVX R ≤13	MEM R ≤19	IPM R ≤19	
VN-3378	E-NS-sw	2010	1	≤2	0.015	**>4**	≤2	≤0.06	2	8	≤4	**32**	≤0.25	≤1	1	26	30	30	30	38	32	(STR)
VN-3379	E-NS-sw	2010	1	≤2	0.015	**>4**	≤2	≤0.06	2	≤4	≤4	16	≤0.25	≤1	1	27	29	30	32	34	30	susceptible
VN-3398	E-NS-sw	2010	1	≤2	0.03	**>4**	≤2	≤0.06	2	16	≤4	**32**	≤0.25	≤1	2	28	31	28	34	34	32	(STR)
VN-3403	E-NS-sw	2010	1	≤2	0.015	**>4**	≤2	≤0.06	2	8	≤4	16	≤0.25	≤1	1	26	32	32	36	36	32	susceptible
VN-3408	E-NS-sw	2010	1	≤2	0.015	**>4**	≤2	≤0.06	2	16	≤4	**32**	≤0.25	≤1	1	30	35	30	34	40	35	(STR)
VN-3410	E-NS-sw	2010	1	≤2	0.015	**>4**	≤2	≤0.06	2	8	≤4	**32**	≤0.25	≤1	1	28	31	28	34	36	36	(STR)
VN-3412	E-NS-sw	2010	1	≤2	0.015	**>4**	≤2	≤0.06	2	8	≤4	16	≤0.25	≤1	1	27	30	28	30	34	32	susceptible
VN-3415	E-NS-sw	2010	1	≤2	0.03	**>4**	≤2	≤0.06	1	8	≤4	**32**	≤0.25	≤1	2	30	34	32	34	40	36	(STR)
VN-3419	E-NS-sw	2010	1	≤2	0.03	**>4**	≤2	≤0.06	2	8	≤4	**32**	0.5	≤1	1	27	30	28	34	36	34	(STR)
VN-3448	E-NS-sw	2010	1	≤2	≤0.008	**>4**	≤2	≤0.06	1	≤4	≤4	16	≤0.25	≤1	≤0.5	29	32	36	36	36	35	susceptible
VN-3451	E-NS-sw	2010	1	≤2	0.015	**>4**	≤2	≤0.06	1	8	≤4	16	≤0.25	≤1	≤0.5	28	32	28	31	34	34	susceptible
VN-3461	E-NS-sw	2010	1	≤2	≤0.008	**>4**	≤2	≤0.06	2	8	≤4	**32**	≤0.25	≤1	≤0.5	30	34	34	40	38	34	(STR)
VN-3477	E-NS-sw	2010	1	≤2	0.015	**>4**	≤2	≤0.06	2	8	≤4	16	≤0.25	≤1	1	26	30	28	34	36	34	susceptible
VN-3494	E-NS-sw	2010	1	≤2	0.015	**>4**	≤2	≤0.06	2	≤4	≤4	16	≤0.25	≤1	1	30	32	32	36	36	34	susceptible
VN-3496	E-NS-sw	2010	1	≤2	0.015	**>4**	≤2	≤0.06	1	8	≤4	16	≤0.25	≤1	≤0.5	29	35	32	38	34	34	susceptible
VN-3506	E-NS-sw	2010	1	≤2	0.03	**>4**	≤2	≤0.06	2	16	≤4	**32**	≤0.25	≤1	1	30	30	30	36	36	36	(STR)
VN-2813	E-NS-sw	2011	1	≤2	0.015	**>4**	≤2	≤0.06	2	8	≤4	**32**	≤0.25	≤1	1	28	34	34	38	36	34	(STR)
VN-2814	E-NS-sw	2011	1	≤2	0.015	**>4**	≤2	≤0.06	2	16	≤4	16	≤0.25	≤1	1	26	28	28	34	34	34	susceptible
VN-3518	E-NS-sw	2012	1	≤2	0.03	**>4**	≤2	≤0.06	2	8	≤4	16	≤0.25	≤1	≤0.5	28	30	28	34	34	32	susceptible
VN-3529	E-NS-sw	2012	1	≤2	0.03	**>4**	≤2	≤0.06	2	8	≤4	16	≤0.25	≤1	1	28	32	30	36	34	32	susceptible
VN-3533	E-NS-sw	2012	1	≤2	0.015	**>4**	≤2	≤0.06	2	8	≤4	16	≤0.25	≤1	1	26	29	30	32	34	32	susceptible
VN-3536	E-NS-sw	2012	1	≤2	0.015	**>4**	≤2	≤0.06	2	8	≤4	**32**	≤0.25	≤1	1	28	34	32	36	40	36	(STR)
VN-3538	E-NS-sw	2012	1	≤2	0.03	**>4**	≤2	≤0.06	2	16	≤4	**32**	≤0.25	≤1	1	28	30	30	32	36	34	(STR)
VN-3539	E-NS-sw	2012	2	≤2	0.015	**>4**	≤2	≤0.06	4	8	≤4	**32**	≤0.25	≤1	≤0.5	26	30	30	34	34	30	(STR)
VN-3541	E-NS-sw	2012	1	≤2	0.015	**>4**	≤2	≤0.06	1	≤4	≤4	**32**	≤0.25	≤1	1	28	33	30	38	38	34	(STR)
VN-3542	E-NS-sw	2012	2	≤2	0.03	**>4**	≤2	0.12	2	8	≤4	**32**	≤0.25	≤1	1	27	30	26	34	34	34	(STR)

AMC, amoxicillin/clavulanic acid; AMP, ampicillin; CAZ, ceftazidime; CHL, chloramphenicol; CIP, ciprofloxacin; CST, colistin; CTX, cefotaxime; FEP, cefepime; FFN, florfenicol; GEN, gentamicin; IPM, imipenem; KAN, kanamycin; LVX, levofloxacin; MEM, meropenem; NAL, nalidixic acid; STR, streptomycin; SXT, trimethoprim/sulfamethoxazole; TET, tetracycline; TMP, trimethoprim.

[a] MIC values or inhibition zone diameters rated as resistant are shown in bolt and red, while those rated as intermediate resistant are shown in bolt.

[b] Strain source code is explained in Table 1.

[c] Resistance profile includes intermediate resistance shown in brackets.

Supplementary Table S2. Results of broth microdilution and disk diffusion assays of all *V. cholerae* isolates[a]

	Strain source code[b]	Year	Minimal inhibitory concentration (MIC) [mg/L]													Inhibition zone diameter [mm]						Resistance Profile[c]
			AMP R ≥32	CHL R ≥32	CIP R ≥4	CST R >2	FFN R ≥16	CTX R ≥4	GEN R ≥16	KAN R ≥64	NAL R ≥32	STR R ≥64	CAZ R ≥16	TET R ≥16	TMP R ≥16	AMC R ≤13	SXT R ≤10	FEP R ≤18	LVX R ≤13	MEM R ≤19	IPM R ≤19	
**Clinical**																						
VN-00297	C-G/A-ext	1995	2	≤2	≤0.008	**>4**	≤2	≤0.06	2	8	≤4	**32**	≤0.25	≤1	≤0.5	23	30	36	19	34	28	(STR)
VN-00298	C-G/A-ext	1995	2	≤2	≤0.008	**>4**	≤2	≤0.06	0.5	≤4	≤4	8	≤0.25	≤1	≤0.5	22	26	34	38	32	26	susceptible
VN-00301	C-G/A-ext	2000	2	≤2	≤0.008	≤2	≤2	≤0.06	1	≤4	≤4	**32**	≤0.25	≤1	1	28	34	40	36	36	36	(STR)
VN-00313	C-G/A-ext	2000	**>32**	≤2	≤0.008	**>4**	≤2	≤0.06	0.5	8	≤4	16	≤0.25	≤1	≤0.5	22.5	28	30	30	26	28	AMP
VN-00314	C-G/A-ext	2005	**>32**	≤2	≤0.008	**>4**	≤2	≤0.06	1	≤4	≤4	16	≤0.25	≤1	≤0.5	20	27	30	36	24	25	AMP
VN-00168	C-G/A-ext	2010	2	≤2	≤0.008	**>4**	≤2	≤0.06	1	≤4	≤4	16	≤0.25	≤1	1	19	25	32	36	24	26	susceptible
VN-00169	C-G/A-ext	2010	4	≤2	≤0.008	**>4**	≤2	≤0.06	0.5	≤4	≤4	16	≤0.25	≤1	1	18	22	30	36	26	26	susceptible
VN-00305	C-G/A-ext	2012	**>32**	≤2	≤0.008	**>4**	≤2	≤0.06	0.5	≤4	≤4	16	≤0.25	≤1	≤0.5	20	24	30	28	26	24	AMP
VN-00307	C-G/A-ext	2012	2	≤2	≤0.008	**>4**	≤2	≤0.06	0.5	≤4	≤4	8	≤0.25	≤1	≤0.5	20	26	26	34	24	26	susceptible
VN-00300	C-G/A-int	1999	4	≤2	≤0.008	**>4**	≤2	≤0.06	0.5	≤4	≤4	16	≤0.25	≤1	≤0.5	18	27	30	34	32	24	susceptible
VN-00302	C-G/A-int	2012	2	≤2	≤0.008	**>4**	≤2	≤0.06	1	≤4	≤4	16	≤0.25	≤1	≤0.5	21	25	32	29	28	28	susceptible
VN-00303	C-ta-ext	2012	≤0.5	≤2	≤0.008	**>4**	≤2	≤0.06	1	8	≤4	**32**	≤0.25	≤1	≤0.5	27	25	34	34	30	28	(STR)
VN-00299	C-ta-int	1996	2	≤2	≤0.008	**>4**	≤2	≤0.06	1	≤4	≤4	16	≤0.25	≤1	≤0.5	22	26	32	38	30	28	susceptible
VN-00210	C-ta-int	2011	2	≤2	≤0.008	**>4**	≤2	≤0.06	1	≤4	≤4	16	≤0.25	≤1	≤0.5	18	25	28	34	24	26	susceptible
VN-00211	C-ta-int	2011	2	≤2	0.5	**>4**	≤2	≤0.06	1	≤4	**>64**	16	≤0.25	≤1	≤0.5	18	27	30	25	25	25	NAL
VN-00315	C-ta-int	2011	2	≤2	≤0.008	**>4**	≤2	≤0.06	1	≤4	≤4	16	≤0.25	≤1	≤0.5	19	27	30	34	26	25	susceptible
VN-00304	C-ta-int	2012	2	≤2	0.5	**>4**	≤2	≤0.06	0.5	≤4	**>64**	16	≤0.25	≤1	≤0.5	22	25	30	24	30	26	NAL
VN-00308	C-ta-int	2012	2	≤2	≤0.008	**>4**	≤2	≤0.06	1	≤4	≤4	16	≤0.25	≤1	1	18	25	28	36	24	26	susceptible
**Environmental Baltic Sea**																						
VN-03916	E-BS-sd	2011	4	≤2	≤0.008	**>4**	≤2	≤0.06	2	≤4	≤4	16	≤0.25	≤1	≤0.5	21	29	34	36	28	25	susceptible
VN-03939	E-BS-sd	2011	2	≤2	≤0.008	**>4**	≤2	≤0.06	1	≤4	≤4	16	≤0.25	≤1	2	22	29	34	34	30	26	susceptible
VN-03940	E-BS-sd	2011	4	≤2	≤0.008	**>4**	≤2	≤0.06	1	≤4	≤4	16	≤0.25	≤1	1	21	31	30	38	30	26	susceptible
VN-03955	E-BS-sd	2011	**>32**	≤2	≤0.008	**>4**	≤2	≤0.06	1	8	≤4	16	≤0.25	≤1	1	18	26	28	30	26	24	AMP
VN-00167	E-BS-sw	2010	2	≤2	≤0.008	**>4**	≤2	≤0.06	0.5	≤4	≤4	16	≤0.25	≤1	≤0.5	18	25	30	34	26	24	susceptible
VN-00478	E-BS-sw	2010	2	≤2	≤0.008	**>4**	≤2	≤0.06	0.5	≤4	≤4	8	≤0.25	≤1	≤0.5	20	26	33	32	26	34	susceptible
VN-03963	E-BS-sw	2010	4	≤2	≤0.008	**>4**	≤2	≤0.06	1	≤4	≤4	16	≤0.25	≤1	≤0.5	21	26	30	22	25	25	susceptible
VN-03967	E-BS-sw	2010	2	≤2	≤0.008	**>4**	≤2	≤0.06	0.5	≤4	≤4	16	≤0.25	≤1	≤0.5	20	26	30	30	26	25	susceptible
VN-00278	E-BS-sw	2011	2	≤2	≤0.008	**>4**	≤2	≤0.06	0.5	≤4	≤4	16	≤0.25	≤1	≤0.5	22	27	34	20	30	28	susceptible
VN-00455	E-BS-sw	2011	2	≤2	≤0.008	**>4**	≤2	≤0.06	1	8	≤4	16	≤0.25	≤1	≤0.5	20	27	32	38	26	25	susceptible
VN-02995	E-BS-sw	2011	2	≤2	≤0.008	**>4**	≤2	≤0.06	1	≤4	≤4	16	≤0.25	≤1	≤0.5	21	28	32	32	28	26	susceptible
VN-02997	E-BS-sw	2011	**>32**	≤2	≤0.008	**>4**	≤2	0.12	≤0.25	≤4	≤4	8	0.5	≤1	≤0.5	**14**	28	28	36	**20**	**14**	AMP, IPM (AMC, MEM)
VN-03903	E-BS-sw	2011	2	≤2	≤0.008	**>4**	≤2	≤0.06	2	≤4	≤4	16	≤0.25	≤1	1	21	27	32	34	29	25	susceptible
VN-03908	E-BS-sw	2011	4	≤2	≤0.008	**>4**	≤2	≤0.06	1	≤4	≤4	16	≤0.25	≤1	1	20	29	30	34	30	24	susceptible
VN-03911	E-BS-sw	2011	2	≤2	≤0.008	**>4**	≤2	≤0.06	1	8	≤4	16	≤0.25	≤1	1	22	27	34	40	30	27	susceptible
VN-03917	E-BS-sw	2011	4	≤2	≤0.008	**>4**	≤2	≤0.06	1	8	≤4	**32**	≤0.25	≤1	1	21	27	32	34	28	25	(STR)
VN-05169	E-BS-sw	2011	4	≤2	≤0.008	**>4**	≤2	≤0.06	2	≤4	≤4	**32**	≤0.25	≤1	1	19	26	26	30	26	26	(STR)

Table continued																						
	Strain source code[b]	Year	Minimal inhibitory concentration (MIC) [mg/L]													Inhibition zone diameter [mm]						Resistance Profile[c]
			AMP R ≥32	CHL R ≥32	CIP R ≥4	CST R >2	FFN R ≥16	CTX R ≥4	GEN R ≥16	KAN R ≥64	NAL R ≥32	STR R ≥64	CAZ R ≥16	TET R ≥16	TMP R ≥16	AMC R ≤13	SXT R ≤10	FEP R ≤18	LVX R ≤13	MEM R ≤19	IPM R ≤19	
VN-05170	E-BS-sw	2011	2	≤2	≤0.008	**>4**	≤2	≤0.06	1	≤4	≤4	**32**	≤0.25	≤1	1	21	26	30	32	28	27	(STR)
VN-05171	E-BS-sw	2011	4	≤2	≤0.008	**>4**	≤2	≤0.06	1	≤4	≤4	16	≤0.25	≤1	1	19	26	32	34	27	24	susceptible
VN-05172	E-BS-sw	2011	4	≤2	≤0.008	**>4**	≤2	≤0.06	1	≤4	≤4	16	≤0.25	≤1	1	21	22	30	34	28	26	susceptible
VN-05173	E-BS-sw	2011	8	≤2	≤0.008	**>4**	≤2	≤0.06	1	≤4	≤4	16	≤0.25	≤1	≤0.5	21	28	30	34	28	28	susceptible
VN-05174	E-BS-sw	2011	**>32**	≤2	≤0.008	**>4**	≤2	≤0.06	2	≤4	≤4	16	≤0.25	≤1	1	20	26	30	36	28	25	AMP
VN-05175	E-BS-sw	2011	2	≤2	≤0.008	**>4**	≤2	≤0.06	1	≤4	≤4	16	≤0.25	≤1	≤0.5	21	27	30	36	30	28	susceptible
VN-05176	E-BS-sw	2011	4	≤2	≤0.008	**>4**	≤2	≤0.06	2	≤4	≤4	16	≤0.25	≤1	1	22	27	32	34	34	25	susceptible
VN-05177	E-BS-sw	2011	2	≤2	≤0.008	**>4**	≤2	≤0.06	1	≤4	≤4	16	≤0.25	≤1	≤0.5	21	30	32	36	30	25	susceptible
VN-05178	E-BS-sw	2011	2	≤2	≤0.008	**>4**	≤2	≤0.06	1	≤4	≤4	**32**	≤0.25	≤1	1	22	29	34	36	32	27	(STR)
VN-05183	E-BS-sw	2011	4	≤2	≤0.008	**>4**	≤2	≤0.06	1	≤4	≤4	16	≤0.25	≤1	≤0.5	20	23	32	34	28	25	susceptible
VN-05184	E-BS-sw	2011	4	≤2	≤0.008	**>4**	≤2	≤0.06	1	≤4	≤4	16	≤0.25	≤1	≤0.5	20	27	31	36	30	28	susceptible
VN-05185	E-BS-sw	2011	2	≤2	≤0.008	**>4**	≤2	≤0.06	1	≤4	≤4	16	≤0.25	≤1	≤0.5	20	27	32	36	30	26	susceptible
VN-05187	E-BS-sw	2011	4	≤2	≤0.008	**>4**	≤2	≤0.06	1	8	≤4	16	≤0.25	≤1	≤0.5	20	28	32	36	27	**22**	(IPM)
VN-00456	E-BS-sw	2012	2	≤2	≤0.008	**>4**	≤2	≤0.06	2	8	≤4	16	≤0.25	≤1	≤0.5	20	26	30	34	27	24	susceptible
VN-00457	E-BS-sw	2012	2	≤2	≤0.008	**>4**	≤2	≤0.06	0.5	≤4	≤4	16	≤0.25	≤1	≤0.5	24	27	32	30	30	25	susceptible
VN-00458	E-BS-sw	2012	4	≤2	≤0.008	**>4**	≤2	≤0.06	0.5	≤4	≤4	**32**	≤0.25	≤1	≤0.5	19	28	30	32	27	24	(STR)
VN-00459	E-BS-sw	2012	2	≤2	≤0.008	**>4**	≤2	≤0.06	1	≤4	≤4	8	≤0.25	≤1	1	20	27	32	34	28	26	susceptible
VN-00460	E-BS-sw	2012	2	≤2	≤0.008	**>4**	≤2	≤0.06	1	≤4	≤4	16	≤0.25	≤1	≤0.5	21	27	30	32	26	24	susceptible
VN-00461	E-BS-sw	2012	4	≤2	≤0.008	**>4**	≤2	≤0.06	0.5	≤4	≤4	16	≤0.25	≤1	≤0.5	20	26	30	30	26	23	susceptible
VN-00462	E-BS-sw	2012	4	≤2	≤0.008	**>4**	≤2	≤0.06	1	≤4	≤4	16	≤0.25	≤1	≤0.5	21	28	30	32	28	24	susceptible
VN-05300	E-BS-sw	2012	2	≤2	≤0.008	**>4**	≤2	≤0.06	2	8	≤4	16	≤0.25	≤1	≤0.5	22	27	32	30	30	24	susceptible
VN-05301	E-BS-sw	2012	4	≤2	≤0.008	**>4**	≤2	≤0.06	1	≤4	≤4	**32**	≤0.25	≤1	≤0.5	21	27	30	34	28	24	(STR)
VN-00463	E-BS-sw	2013	2	≤2	≤0.008	**>4**	≤2	≤0.06	2	≤4	≤4	16	≤0.25	≤1	1	20	27	32	30	28	28	susceptible
VN-00464	E-BS-sw	2013	4	≤2	≤0.008	**>4**	≤2	≤0.06	1	≤4	≤4	16	≤0.25	≤1	≤0.5	21	27	32	34	28	24	susceptible
VN-00465	E-BS-sw	2013	2	≤2	≤0.008	**>4**	≤2	≤0.06	1	≤4	≤4	**32**	≤0.25	≤1	≤0.5	21	26	30	32	28	24	(STR)
VN-00466	E-BS-sw	2013	2	≤2	≤0.008	**>4**	≤2	≤0.06	1	≤4	≤4	16	≤0.25	≤1	≤0.5	21	27	32	32	30	26	susceptible
VN-00467	E-BS-sw	2013	2	≤2	≤0.008	**>4**	≤2	≤0.06	1	≤4	≤4	16	≤0.25	≤1	1	20	25	30	30	26	24	susceptible
VN-00468	E-BS-sw	2013	2	≤2	≤0.008	**>4**	≤2	≤0.06	1	≤4	≤4	16	≤0.25	≤1	1	20	27	30	30	28	24	susceptible
VN-00469	E-BS-sw	2013	4	≤2	≤0.008	**>4**	≤2	≤0.06	0.5	≤4	≤4	16	≤0.25	≤1	1	21	30	32	34	30	27	susceptible
VN-00470	E-BS-sw	2013	2	≤2	≤0.008	**>4**	≤2	≤0.06	0.5	≤4	≤4	16	≤0.25	≤1	1	20	27	28	30	27	23	susceptible
VN-00471	E-BS-sw	2013	2	≤2	≤0.008	**>4**	≤2	≤0.06	1	≤4	≤4	8	≤0.25	≤1	≤0.5	20	28	30	34	28	28	susceptible
VN-00472	E-BS-sw	2013	2	≤2	≤0.008	**>4**	≤2	≤0.06	2	≤4	≤4	16	≤0.25	≤1	≤0.5	21	22	30	30	28	24	susceptible
VN-00473	E-BS-sw	2013	4	≤2	≤0.008	**>4**	≤2	≤0.06	1	8	≤4	16	≤0.25	≤1	1	20	27	30	30	26	23	susceptible
VN-00474	E-BS-sw	2013	2	≤2	≤0.008	**>4**	≤2	≤0.06	1	≤4	≤4	16	≤0.25	≤1	≤0.5	19	28	30	32	28	26	susceptible
VN-04241	E-BS-sw	2013	2	≤2	≤0.008	**>4**	≤2	≤0.06	1	≤4	≤4	16	≤0.25	≤1	≤0.5	19	26	32	36	26	24	susceptible
VN-04247	E-BS-sw	2013	8	≤2	≤0.008	**>4**	≤2	≤0.06	1	≤4	≤4	**32**	≤0.25	≤1	≤0.5	18	28	30	34	24	**20**	(STR, IPM)
VN-04250	E-BS-sw	2013	2	≤2	≤0.008	**>4**	≤2	≤0.06	2	8	≤4	16	≤0.25	≤1	1	20	25	32	30	28	26	susceptible
VN-04251	E-BS-sw	2013	4	≤2	≤0.008	**>4**	≤2	≤0.06	2	≤4	≤4	**32**	≤0.25	≤1	1	21	27	32	32	28	26	(STR)
VN-00475	E-BS-sw	2014	4	≤2	≤0.008	**>4**	≤2	≤0.06	1	8	≤4	16	≤0.25	≤1	≤0.5	20	27	26	30	30	24	susceptible
VN-00476	E-BS-sw	2014	2	≤2	≤0.008	**>4**	≤2	≤0.06	0.5	8	≤4	16	≤0.25	≤1	1	38	26	30	30	26	23	susceptible

Table continued																						
	Strain source code[b]	Year	Minimal inhibitory concentration (MIC) [mg/L]													Inhibition zone diameter [mm]						Resistance Profile[c]
			AMP R ≥32	CHL R ≥32	CIP R ≥4	CST R >2	FFN R ≥16	CTX R ≥4	GEN R ≥16	KAN R ≥64	NAL R ≥32	STR R ≥64	CAZ R ≥16	TET R ≥16	TMP R ≥16	AMC R ≤13	SXT R ≤10	FEP R ≤18	LVX R ≤13	MEM R ≤19	IPM R ≤19	
VN-00477	E-BS-sw	2014	2	≤2	≤0.008	**>4**	≤2	≤0.06	1	≤4	≤4	16	≤0.25	≤1	≤0.5	20	26	32	36	28	24	susceptible
VN-03901	E-BS-sw/sd	2011	2	≤2	≤0.008	**>4**	≤2	≤0.06	1	8	≤4	**32**	≤0.25	≤1	≤0.5	22	28	32	36	26	26	(STR)
VN-03902	E-BS-sw/sd	2011	2	≤2	≤0.008	**>4**	≤2	≤0.06	2	8	≤4	16	≤0.25	≤1	≤0.5	25	30	34	40	32	30	susceptible
VN-03907	E-BS-sw/sd	2011	2	≤2	≤0.008	**>4**	≤2	≤0.06	1	8	≤4	16	≤0.25	≤1	≤0.5	21	28	34	36	30	26	susceptible
VN-03913	E-BS-sw/sd	2011	4	≤2	≤0.008	**>4**	≤2	≤0.06	2	≤4	≤4	**32**	≤0.25	≤1	1	21	29	34	36	28	26	(STR)
VN-03918	E-BS-sw/sd	2011	4	≤2	≤0.008	**>4**	≤2	≤0.06	2	≤4	≤4	**32**	≤0.25	≤1	1	20	26	34	38	28	26	(STR)
VN-03923	E-BS-sw/sd	2011	2	≤2	≤0.008	**>4**	≤2	≤0.06	1	≤4	≤4	16	≤0.25	≤1	≤0.5	20	28	34	38	26	25	susceptible
VN-03936	E-BS-sw/sd	2011	4	≤2	≤0.008	**>4**	≤2	≤0.06	2	≤4	≤4	16	≤0.25	≤1	≤0.5	20	29	30	34	30	24	susceptible
VN-03938	E-BS-sw/sd	2011	2	≤2	≤0.008	**>4**	≤2	≤0.06	1	≤4	≤4	16	≤0.25	≤1	1	21	28	30	34	32	26	susceptible
VN-03941	E-BS-sw/sd	2011	2	≤2	≤0.008	**>4**	≤2	≤0.06	1	≤4	≤4	16	≤0.25	≤1	≤0.5	19	28	34	34	28	24	susceptible
VN-03942	E-BS-sw/sd	2011	**>32**	≤2	≤0.008	**>4**	≤2	≤0.06	1	8	≤4	16	≤0.25	≤1	≤0.5	19	27	28	32	26	24	AMP
VN-03943	E-BS-sw/sd	2011	2	≤2	≤0.008	**>4**	≤2	≤0.06	1	8	≤4	**32**	≤0.25	≤1	1	21	27	30	32	26	25	(STR)
VN-03944	E-BS-sw/sd	2011	2	≤2	≤0.008	**>4**	≤2	≤0.06	1	8	≤4	**32**	≤0.25	≤1	≤0.5	22	28	28	30	26	25	(STR)
VN-03949	E-BS-sw/sd	2011	4	≤2	≤0.008	**>4**	≤2	≤0.06	1	≤4	≤4	16	≤0.25	≤1	≤0.5	21	27	30	32	28	25	susceptible
VN-03950	E-BS-sw/sd	2011	4	≤2	≤0.008	**>4**	≤2	≤0.06	1	≤4	≤4	16	≤0.25	≤1	≤0.5	21	25	30	32	26	24	susceptible
VN-03951	E-BS-sw/sd	2011	2	≤2	≤0.008	**>4**	≤2	≤0.06	0.5	≤4	≤4	8	≤0.25	≤1	≤0.5	25	27	34	38	30	29	susceptible
VN-03952	E-BS-sw/sd	2011	2	≤2	≤0.008	**>4**	≤2	≤0.06	1	≤4	≤4	16	≤0.25	≤1	≤0.5	22	28	34	34	30	26	susceptible
VN-03953	E-BS-sw/sd	2011	2	≤2	≤0.008	**>4**	≤2	≤0.06	0.5	≤4	≤4	16	≤0.25	≤1	1	21	27	32	34	28	25	susceptible
VN-03954	E-BS-sw/sd	2011	**>32**	≤2	≤0.008	**>4**	≤2	≤0.06	1	≤4	≤4	8	≤0.25	≤1	1	19	26	26	26	24	25	AMP
VN-03956	E-BS-sw/sd	2011	2	≤2	≤0.008	**>4**	≤2	≤0.06	2	≤4	≤4	**32**	≤0.25	≤1	1	22	25	32	32	28	26	(STR)
VN-03957	E-BS-sw/sd	2011	2	≤2	≤0.008	**>4**	≤2	≤0.06	0.5	≤4	≤4	8	≤0.25	≤1	1	28	29	34	36	28	26	susceptible
VN-03958	E-BS-sw/sd	2011	2	≤2	≤0.008	**>4**	≤2	≤0.06	1	≤4	≤4	16	≤0.25	≤1	1	23	28	32	34	29	28	susceptible
**Environmental North Sea**																						
VN-10012	E-NS-bm	2011	2	≤2	≤0.008	**>4**	≤2	≤0.06	1	≤4	≤4	16	≤0.25	≤1	≤0.5	18	27	30	34	24	26	susceptible
VN-10013	E-NS-bm	2011	2	≤2	≤0.008	**>4**	≤2	≤0.06	0.5	≤4	≤4	16	≤0.25	≤1	≤0.5	18	27	30	34	24	26	susceptible
VN-10127	E-NS-bm	2012	**>32**	≤2	≤0.008	**>4**	≤2	≤0.06	1	8	≤4	16	≤0.25	≤1	≤0.5	21	28	32	30	26	25	AMP
VN-10130	E-NS-bm	2012	**>32**	≤2	≤0.008	**>4**	≤2	≤0.06	2	≤4	≤4	16	≤0.25	≤1	1	19	27	30	30	28	26	AMP
VN-10131	E-NS-bm	2012	**>32**	≤2	≤0.008	**>4**	≤2	≤0.06	1	≤4	≤4	16	≤0.25	≤1	1	21	27	30	31	28	25	AMP
VN-10133	E-NS-bm	2012	2	≤2	≤0.008	**>4**	≤2	≤0.06	1	≤4	≤4	16	≤0.25	≤1	≤0.5	21	28	32	36	30	28	susceptible
VN-10137	E-NS-bm	2012	4	≤2	≤0.008	**>4**	≤2	≤0.06	2	8	≤4	**32**	≤0.25	≤1	1	20	28	34	32	28	34	(STR)
VN-10143	E-NS-bm	2012	4	≤2	≤0.008	**4**	≤2	≤0.06	2	16	≤4	16	≤0.25	≤1	≤0.5	21	32	34	36	30	27	susceptible
VN-10144	E-NS-bm	2012	4	≤2	≤0.008	≤2	≤2	≤0.06	2	≤4	≤4	**32**	≤0.25	≤1	1	25	34	34	46	30	28	(STR)
VN-10145	E-NS-bm	2012	2	≤2	≤0.008	≤2	≤2	≤0.06	4	8	≤4	**32**	≤0.25	≤1	≤0.5	23	34	34	42	34	30	(STR)
VN-10146	E-NS-bm	2012	2	≤2	≤0.008	≤2	≤2	≤0.06	1	≤4	≤4	16	≤0.25	≤1	1	25	34	50	50	40	28	susceptible
VN-10150	E-NS-bm	2012	4	≤2	≤0.008	**>4**	≤2	≤0.06	1	≤4	≤4	16	≤0.25	≤1	1	19	27	32	36	26	24	susceptible
VN-10156	E-NS-bm	2012	2	≤2	≤0.008	**>4**	≤2	≤0.06	1	≤4	≤4	16	≤0.25	≤1	≤0.5	21	28	32	30	30	24	susceptible
VN-10159	E-NS-bm	2012	2	≤2	≤0.008	**>4**	≤2	≤0.06	1	≤4	≤4	16	≤0.25	≤1	≤0.5	20	29	31	32	28	24	susceptible
VN-10162	E-NS-bm	2012	4	≤2	≤0.008	**>4**	≤2	≤0.06	1	8	≤4	16	≤0.25	≤1	≤0.5	19	27	28	32	28	24	susceptible
VN-10191	E-NS-bm	2013	4	≤2	≤0.008	**>4**	≤2	≤0.06	4	8	≤4	**64**	≤0.25	≤1	≤0.5	20	28	31	36	28	25	STR
VN-10192	E-NS-bm	2013	4	≤2	≤0.008	**>4**	≤2	≤0.06	2	16	≤4	**64**	≤0.25	≤1	≤0.5	20	30	33	40	32	28	STR

Table continued																						
	Strain source code[b]	Year	Minimal inhibitory concentration (MIC) [mg/L]													Inhibition zone diameter [mm]						Resistance Profile[c]
			AMP R ≥32	CHL R ≥32	CIP R ≥4	CST R >2	FFN R ≥16	CTX R ≥4	GEN R ≥16	KAN R ≥64	NAL R ≥32	STR R ≥64	CAZ R ≥16	TET R ≥16	TMP R ≥16	AMC R ≤13	SXT R ≤10	FEP R ≤18	LVX R ≤13	MEM R ≤19	IPM R ≤19	
VN-10196	E-NS-bm	2013	2	≤2	0.06	**>4**	≤2	≤0.06	1	16	≤4	16	≤0.25	≤1	≤0.5	22	29	34	30	28	25	susceptible
VN-10197	E-NS-bm	2013	2	≤2	≤0.008	**>4**	≤2	≤0.06	1	8	≤4	16	≤0.25	≤1	≤0.5	20	26	32	36	26	25	susceptible
VN-10198	E-NS-bm	2013	4	≤2	≤0.008	**>4**	≤2	≤0.06	1	≤4	≤4	16	≤0.25	≤1	≤0.5	21	27	32	34	27	25	susceptible
VN-10204	E-NS-bm	2013	2	≤2	≤0.008	**>4**	≤2	≤0.06	0.5	≤4	≤4	16	≤0.25	≤1	≤0.5	24	28	34	36	30	28	susceptible
VN-10205	E-NS-bm	2013	2	≤2	≤0.008	**>4**	≤2	≤0.06	4	8	≤4	**32**	≤0.25	≤1	≤0.5	23	29	34	34	30	27	(STR)
VN-10206	E-NS-bm	2013	2	≤2	≤0.008	**>4**	≤2	≤0.06	1	8	≤4	8	≤0.25	≤1	≤0.5	23	29	34	36	30	28	susceptible
VN-10207	E-NS-bm	2013	2	≤2	≤0.008	**>4**	≤2	≤0.06	2	≤4	≤4	**32**	≤0.25	≤1	≤0.5	23	28	34	34	34	27	(STR)
VN-10208	E-NS-bm	2013	2	≤2	≤0.008	**>4**	≤2	≤0.06	2	≤4	≤4	16	≤0.25	≤1	1	23	29	32	36	30	29	susceptible
VN-10320	E-NS-bm	2014	2	≤2	0.06	**>4**	≤2	≤0.06	1	8	≤4	16	≤0.25	≤1	≤0.5	24	29	36	30	32	28	susceptible
VN-02808	E-NS-sw	2011	**>32**	≤2	≤0.008	**>4**	≤2	≤0.06	1	≤4	≤4	**32**	≤0.25	≤1	≤0.5	**15**	29	28	35	**19**	**15**	AMP, IPM, MEM (AMC, STR)
VN-02825	E-NS-sw	2011	**>32**	≤2	≤0.008	**>4**	≤2	≤0.06	0.5	≤4	≤4	8	0.5	≤1	≤0.5	**14**	30	26	34	**20**	**15**	AMP, IPM (AMC, MEM)
VN-02923	E-NS-sw	2011	**>32**	≤2	≤0.008	**>4**	≤2	≤0.06	0.5	≤4	≤4	16	≤0.25	≤1	≤0.5	**14**	30	28	26	**20**	**16**	AMP, IPM (AMC, MEM)
VN-04216	E-NS-sw	2013	2	≤2	≤0.008	**>4**	≤2	≤0.06	1	8	≤4	16	≤0.25	≤1	≤0.5	21	27	31	32	28	26	susceptible
VN-04219	E-NS-sw	2013	2	≤2	≤0.008	**>4**	≤2	≤0.06	1	≤4	≤4	16	≤0.25	≤1	≤0.5	22	28	28	34	32	26	susceptible
VN-04223	E-NS-sw	2013	2	≤2	≤0.008	**>4**	≤2	≤0.06	1	≤4	≤4	16	≤0.25	≤1	≤0.5	21	28	32	32	30	28	susceptible
VN-04226	E-NS-sw	2013	**>32**	≤2	≤0.008	**>4**	≤2	≤0.06	1	≤4	≤4	8	≤0.25	≤1	≤0.5	22	30	32	34	30	26	AMP
VN-04231	E-NS-sw	2013	2	≤2	≤0.008	**>4**	≤2	≤0.06	1	≤4	≤4	16	≤0.25	≤1	≤0.5	23	30	32	36	32	30	susceptible
VN-04233	E-NS-sw	2013	8	≤2	≤0.008	**>4**	≤2	≤0.06	0.5	≤4	≤4	16	≤0.25	≤1	≤0.5	20	27	30	30	28	24	susceptible
VN-04261	E-NS-sw	2013	**>32**	≤2	≤0.008	**>4**	≤2	≤0.06	1	≤4	≤4	**32**	≤0.25	≤1	≤0.5	20	28	30	34	28	25	AMP (STR)
VN-03213	E-NS-sw	2014	2	≤2	≤0.008	**>4**	≤2	≤0.06	2	16	≤4	**32**	≤0.25	≤1	1	18	28	30	34	28	25	(STR)
VN-03012	E-NS-sw	2009 - 2011	4	≤2	≤0.008	**>4**	≤2	≤0.06	1	≤4	≤4	16	0.5	≤1	≤0.5	19	29	32	32	28	24	susceptible
VN-03503	E-NS-sw/sd	2009	2	≤2	≤0.008	**>4**	≤2	≤0.06	0.5	≤4	≤4	8	≤0.25	≤1	2	24	27	34	34	30	28	susceptible
VN-03301	E-NS-sw/sd	2010	4	≤2	≤0.008	**>4**	≤2	≤0.06	1	≤4	≤4	16	≤0.25	≤1	≤0.5	19	32	32	34	26	25	susceptible
VN-03361	E-NS-sw/sd	2010	2	≤2	≤0.008	**>4**	≤2	≤0.06	1	8	≤4	**32**	≤0.25	≤1	≤0.5	20	27	30	34	26	26	(STR)
VN-03377	E-NS-sw/sd	2010	2	≤2	≤0.008	**>4**	≤2	≤0.06	1	8	≤4	16	≤0.25	≤1	1	20	28	31	38	28	26	susceptible
VN-03405	E-NS-sw/sd	2010	4	≤2	≤0.008	**>4**	≤2	≤0.06	2	≤4	≤4	**32**	≤0.25	≤1	≤0.5	19	22	30	30	26	25	(STR)
VN-03407	E-NS-sw/sd	2010	4	≤2	≤0.008	**>4**	≤2	≤0.06	0.5	≤4	≤4	8	≤0.25	≤1	≤0.5	19	28	30	32	26	24	susceptible
VN-03428	E-NS-sw/sd	2010	1	≤2	≤0.008	**>4**	≤2	≤0.06	2	≤4	≤4	**32**	≤0.25	≤1	1	24	26	32	34	28	28	(STR)
VN-03460	E-NS-sw/sd	2010	4	≤2	≤0.008	**>4**	≤2	≤0.06	2	8	≤4	16	≤0.25	≤1	≤0.5	20	28	32	34	26	25	susceptible
VN-03469	E-NS-sw/sd	2010	4	≤2	≤0.008	**>4**	≤2	≤0.06	1	8	≤4	**64**	≤0.25	≤1	≤0.5	20	26	30	34	30	24	STR
VN-03470	E-NS-sw/sd	2010	4	≤2	≤0.008	**>4**	≤2	≤0.06	2	8	≤4	**32**	0.5	≤1	≤0.5	20	28	30	40	24	25	(STR)
VN-03471	E-NS-sw/sd	2010	2	≤2	≤0.008	**>4**	≤2	≤0.06	0.5	≤4	≤4	16	≤0.25	≤1	≤0.5	19	28	30	32	26	25	susceptible
VN-03472	E-NS-sw/sd	2010	4	≤2	≤0.008	**>4**	≤2	≤0.06	4	≤4	≤4	**32**	≤0.25	≤1	≤0.5	20	28	30	32	28	24	(STR)
VN-03475	E-NS-sw/sd	2010	4	≤2	≤0.008	**>4**	≤2	≤0.06	1	8	≤4	**32**	≤0.25	≤1	≤0.5	20	28	30	38	28	24	(STR)
VN-03492	E-NS-sw/sd	2011	4	≤2	≤0.008	**>4**	≤2	≤0.06	1	≤4	≤4	**32**	≤0.25	≤1	≤0.5	20	28	32	36	26	25	(STR)

Table continued

	Strain source code[b]	Year	Minimal inhibitory concentration (MIC) [mg/L]													Inhibition zone diameter [mm]						Resistance Profile[c]
			AMP R ≥32	CHL R ≥32	CIP R ≥4	CST R >2	FFN R ≥16	CTX R ≥4	GEN R ≥16	KAN R ≥64	NAL R ≥32	STR R ≥64	CAZ R ≥16	TET R ≥16	TMP R ≥16	AMC R ≤13	SXT R ≤10	FEP R ≤18	LVX R ≤13	MEM R ≤19	IPM R ≤19	
**Retail**																						
VN-05008	R-G-bm	2009	4	≤2	≤0.008	**>4**	≤2	≤0.06	1	8	≤4	16	≤0.25	≤1	1	21	28	32	32	30	26	susceptible
VN-05010	R-G-bm	2009	2	≤2	≤0.008	**>4**	≤2	≤0.06	1	16	≤4	16	≤0.25	≤1	1	22	26	32	33	28	24	susceptible
VN-00014	R-G-cr	2008	2	≤2	≤0.008	**>4**	≤2	≤0.06	1	≤4	≤4	16	≤0.25	≤1	1	20	26	30	34	29	27	susceptible
VN-00015	R-G-cr	2008	2	≤2	≤0.008	**>4**	≤2	≤0.06	1	≤4	≤4	16	≤0.25	≤1	≤0.5	19	25	30	38	24	26	susceptible
VN-00161	R-G-cr	2009	2	≤2	≤0.008	**>4**	≤2	≤0.06	1	≤4	≤4	8	≤0.25	≤1	≤0.5	18	29	30	38	28	26	susceptible
VN-05066	R-G-cr	2011	2	≤2	≤0.008	**>4**	≤2	≤0.06	2	≤4	≤4	16	≤0.25	≤1	≤0.5	21	27	32	32	28	25	susceptible
VN-05095	R-G-cr	2011	2	≤2	≤0.008	**>4**	≤2	≤0.06	4	16	≤4	**64**	≤0.25	≤1	≤0.5	21	25	30	30	28	28	STR
VN-05096	R-G-cr	2011	2	≤2	≤0.008	**4**	≤2	≤0.06	1	≤4	≤4	16	≤0.25	≤1	≤0.5	22	28	35	32	30	28	susceptible
VN-05102	R-G-cr	2011	2	≤2	0.06	**>4**	≤2	≤0.06	0.5	8	≤4	16	≤0.25	≤1	≤0.5	21	27	32	26	26	23	susceptible
VN-05109	R-G-cr	2011	**>32**	≤2	≤0.008	**>4**	≤2	≤0.06	2	≤4	≤4	**32**	≤0.25	≤1	≤0.5	19	27	28	34	28	24	AMP (STR)
VN-05221	R-G-cr	2011	2	≤2	≤0.008	**>4**	≤2	≤0.06	1	≤4	≤4	16	≤0.25	≤1	≤0.5	20	27	32	36	27	25	susceptible
VN-05222	R-G-cr	2011	2	≤2	≤0.008	**>4**	≤2	≤0.06	1	8	≤4	**32**	≤0.25	≤1	≤0.5	19	26	30	32	27	23	(STR)
VN-05223	R-G-cr	2011	2	≤2	≤0.008	**>4**	≤2	≤0.06	1	≤4	≤4	16	≤0.25	≤1	≤0.5	20	26	30	36	24	25	susceptible
VN-00434	R-G-cr	2012	2	≤2	≤0.008	**>4**	≤2	≤0.06	1	8	≤4	16	≤0.25	≤1	≤0.5	18	25	30	34	24	27	susceptible
VN-00435	R-G-cr	2012	2	≤2	≤0.008	**>4**	≤2	≤0.06	1	≤4	≤4	16	≤0.25	≤1	1	21	25	30	34	30	24	susceptible
VN-00436	R-G-cr	2012	4	≤2	≤0.008	**>4**	≤2	≤0.06	1	≤4	≤4	16	≤0.25	≤1	≤0.5	20	27	30	36	26	25	susceptible
VN-00437	R-G-cr	2012	2	≤2	≤0.008	**>4**	≤2	≤0.06	1	≤4	≤4	16	≤0.25	≤1	≤0.5	22	28	32	36	30	25	susceptible
VN-00438	R-G-cr	2012	2	≤2	0.06	**>4**	≤2	≤0.06	1	≤4	16	16	≤0.25	≤1	1	23	27	32	30	27	25	susceptible
VN-00439	R-G-cr	2012	**>32**	≤2	≤0.008	**>4**	≤2	≤0.06	1	≤4	≤4	16	≤0.25	≤1	1	21	27	32	30	26	26	AMP
VN-00440	R-G-cr	2012	4	≤2	≤0.008	**>4**	≤2	≤0.06	1	≤4	≤4	16	≤0.25	≤1	≤0.5	22	29	28	34	28	26	susceptible
VN-00441	R-G-cr	2012	2	≤2	≤0.008	**>4**	≤2	≤0.06	2	8	≤4	16	≤0.25	≤1	≤0.5	21	28	32	34	28	25	susceptible
VN-00442	R-G-cr	2013	2	≤2	≤0.008	**>4**	≤2	≤0.06	2	≤4	≤4	16	≤0.25	≤1	≤0.5	21	29	30	36	28	25	susceptible
VN-05417	R-G-cr	2013	2	≤2	0.03	**>4**	≤2	≤0.06	0.5	≤4	≤4	16	≤0.25	≤1	≤0.5	22	28	32	28	28	28	susceptible
VN-00446	R-G-cr	2014	**>32**	≤2	≤0.008	**>4**	≤2	≤0.06	2	≤4	≤4	16	≤0.25	≤1	≤0.5	20	26	30	33	28	24	AMP
VN-00449	R-G-cr	2014	2	≤2	≤0.008	**>4**	≤2	≤0.06	1	≤4	≤4	16	≤0.25	≤1	1	25	27	30	34	30	30	susceptible
VN-00451	R-G-cr	2014	2	≤2	≤0.008	**>4**	≤2	≤0.06	1	≤4	≤4	16	≤0.25	≤1	≤0.5	21	29	32	34	26	25	susceptible
VN-00452	R-G-cr	2014	2	≤2	≤0.008	**>4**	≤2	≤0.06	1	≤4	≤4	16	≤0.25	≤1	≤0.5	20	26	34	34	26	24	susceptible
VN-00454	R-G-cr	2014	2	≤2	≤0.008	**>4**	≤2	≤0.06	1	≤4	≤4	16	≤0.25	≤1	≤0.5	22	28	34	34	28	25	susceptible
VN-00001	R-G-fi	2008	2	≤2	≤0.008	**>4**	≤2	≤0.06	1	≤4	≤4	16	≤0.25	≤1	1	18	27	30	32	23	26	susceptible
VN-00432	R-G-fi	2012	≤0.5	≤2	≤0.008	**>4**	≤2	≤0.06	1	≤4	≤4	**32**	≤0.25	≤1	≤0.5	24	27	34	38	26	30	(STR)
VN-00433	R-G-fi	2012	1	≤2	≤0.008	**>4**	≤2	≤0.06	1	≤4	≤4	16	≤0.25	≤1	≤0.5	24	26	34	38	28	30	susceptible
VN-00443	R-G-fi	2013	**>32**	≤2	0.06	≤2	≤2	≤0.06	0.5	≤4	16	**32**	≤0.25	≤1	≤0.5	22	30	34	32	32	26	AMP (STR)
VN-00444	R-G-fi	2013	2	≤2	≤0.008	**>4**	≤2	≤0.06	2	≤4	≤4	16	≤0.25	≤1	≤0.5	21	28	34	36	28	25	susceptible
VN-00445	R-G-fi	2013	2	≤2	≤0.008	**>4**	≤2	≤0.06	1	≤4	≤4	16	≤0.25	≤1	1	20	26	30	35	28	24	susceptible
VN-00450	R-G-fi	2014	2	≤2	0.06	**>4**	≤2	≤0.06	0.5	≤4	≤4	16	≤0.25	≤1	**>32**	23	20	36	32	30	26	TMP

AMC, amoxicillin/clavulanic acid; AMP, ampicillin; CAZ, ceftazidime; CHL, chloramphenicol; CIP, ciprofloxacin; CST, colistin; CTX, cefotaxime; FEP, cefepime; FFN, florfenicol; GEN, gentamicin; IPM, imipenem; KAN, kanamycin; LVX, levofloxacin; MEM, meropenem; NAL, nalidixic acid; STR, streptomycin; SXT, trimethoprim/sulfamethoxazole; TET, tetracycline; TMP, trimethoprim.

[a] MIC values or inhibition zone diameters rated as resistant are shown in bolt and red, while those rated as intermediate resistant are shown in bolt.

[b] Strain source code is explained in Table 1.

[c] Resistance profile includes intermediate resistance shown in brackets.

Supplementary Table S3. Interpretive criteria used for broth microdilution and disk diffusion antimicrobial susceptibility testing of *Vibrio* spp.

Antimicrobial Subclass	Antimicrobial agent	Abbre-viation	Test range (mg/L)	MIC breakpoint (mg/L)			Disk content (µg)	Zone diameter breakpoint (mm)			Reference
				S	I	R		S	I	R	
**β-Lactams**											
Aminopenicillins	Ampicillin	AMP	0.5-32	≤8	16	≥32	10	≥17	14-16	≤13	M45-A2, (CLSI, 2010a)
Aminopenicillins/β-lactamase inhibitors	Amoxicillin/ Clavulanic Acid	AMC	-	≤8/4	16/8	≥32/16	20/10	≥18	14-17	≤13	M45-A2, (CLSI, 2010a)
Penicillinase-stable penicillins	Temocillin	TMC	0.5-128	≤ 8		>8	-	-	-	-	(Andrews, 2009)
3rd Gen. cephalosporins	Cefotaxime	CTX	0.06-4	≤1	2	≥4	30	≥26	23-25	≤22	M45-A2, (CLSI, 2010a)
	Ceftazidime	CAZ	0.25-16	≤4	8	≥16	30	≥21	18-20	≤17	M45-A2, (CLSI, 2010a)
4th Gen. cephalosporins	Cefepime	FEP	0.06-32	≤2	4-8[a]	≥16	30	≥25	19-24 [a]	≤18	M100-S25, (CLSI, 2015)
Cephamycins	Cefoxitin	FOX	0.5-64	≤8	16	≥32	30	≥18	15-17	≤14	M45-A2, (CLSI, 2010a)
Carbapenems	Ertapenem	ETP	0.015-2	≤0,5	1	≥2	10	≥22	19-21	≤18	M100-S25, (CLSI, 2015)
	Imipenem	IPM	0.12-16	≤1	2	≥4	10	≥23	20-22	≤19	M100-S25, (CLSI, 2015)
	Meropenem	MEM	-	≤1	2	≥4	10	≥23	20-22	≤19	M100-S25, (CLSI, 2015)
Monobactams	Aztreonam	ATM	-	≤4	8	≥16	30	≥21	18-20	≤17	M100-S25, (CLSI, 2015)
**Non β-Lactams**											
Phenicols	Florfenicol	FFN	2-64	≤4	8	≥16		-	-	-	Vet01-S2, *S. cholerasuis* (CLSI, 2013)
	Chloramphenicol	CHL	2-64	≤8	16	≥32	30	≥18	13-17	≤12	M45-A2, (CLSI, 2010a)
Quinolones	Nalidixic acid	NAL	4-64	≤16	-	≥32	30	≥19	14-18	≤13	M100-S25, (CLSI, 2015)
Fluoroquinolones	Ciprofloxacin	CIP	0.008-8	≤1	2	≥4	5	≥21	16-20	≤15	M45-A2, (CLSI, 2010a)
	Levofloxacin	LVX	-	≤2	4	≥8	5	≥17	14-16	≤13	M45-A2, (CLSI, 2010a)
Aminoglycosides	Gentamicin	GEN	0.25-32	≤4	8	≥16	10	≥15	13-14	≤12	M100-S25, (CLSI, 2015)
	Kanamycin	KAN	4-128	≤16	32	≥64	30	≥18	14-17	≤13	M100-S25, (CLSI, 2015)
	Streptomycin	STR	2-128	≤16	32	≥64	-	-	-	-	(National Food Institute, 2013;Shaw et al., 2014)
Tetracyclines	Tetracycline	TET	1-64	≤4	8	≥16	30	≥15	12-14	≤11	M45-A2, (CLSI, 2010a)
Folate pathway inhibitors	Trimethoprim	TMP	0.5-32	≤8		≥16	5	≥16	11-15	≤10	M100-S25, (CLSI, 2015)
	Sulfamethoxazole / Trimethoprim	SXT	-	≤2/38	-	≥4/76	1.25/23.75	≥16	11-15	≤10	M45-A2, (CLSI, 2010a)
Polymyxins	Colistin	CST	2-4	≤2		>2	-	-	-	-	(EUCAST, 2015)

[a] SDD (susceptible dose-dependent) Breakpoints shown are clinical breakpoints. Clinical breakpoints specific for *Vibrio* spp. described in CLSI document M45-A2 (CLSI, 2010a) derived from breakpoints specific for *Enterobacteriaceae* given in CLSI document M100-S20 (CLSI, 2010b). Subsequent to the approval of document M45-A2 in 2010 carbapenem-specific breakpoints described for *Enterobacteriaceae* were changed (CLSI, 2010c). Thus in case of carbapenems as well as in other cases where specific breakpoints for *Vibrio* spp. were obsolete or not available, latest

breakpoints for *Enterobacteriaceae* were used (CLSI, 2015). Other interpretive criteria were used for colistin (EUCAST clinical breakpoints for *Enterobacteriaceae* (http://www.eucast.org.) (EUCAST, 2015), temocillin (BSAC interpretive criteria for systemic infections) (Andrews, 2009) and streptomycin (based on different studies of *Vibrio* spp. (Shaw et al., 2014) and *E. coli* (National Food Institute, 2013), as no CLSI breakpoints were available.

Supplementary Table 4. Primers used for species identification, characterization and detection of resistance determinants

Gene/Target	Primer name	Sequence (5'to 3')	Amplicon (bp)	T_a (ºC)	Reference
**Species Identification and Characterization**					
toxR	UtoxF	GASTTTGTTTGGCGYGARCAAGGTT		59/60	(Bauer and Roervik, 2007)
toxR of *V. cholerae*	VctoxR	GGTTAGCAACGATGCGTAAG	640	59/60	(Bauer and Roervik, 2007)
toxR of *V. vulnificus*	VvtoxR	AACGGAACTTAGACTCCGAC	435	60	(Bauer and Roervik, 2007)
O139 *rfb*	O139F	AGCCTCTTTATTACGGGTGG	449	59	(Hoshino et al., 1998)
O139 *rfb*	O139R	GTCAAACCCGATCGTAAAGG			(Hoshino et al., 1998)
O1 *rfb*	O1F	GTTTCACTGAACAGATGGG	192		(Hoshino et al., 1998)
O1 *rfb*	O1R	GGTCATCTGTAAGTACAAC			(Hoshino et al., 1998)
ctxA	ctxA1	CTCAGACGGGATTTGTTAGGCACG	301		(Shirai et al., 1991)
ctxA	ctxA2	TCTATCTCTGTAGCCCCTATTACG			(Shirai et al., 1991)
**Detection of Resistance Determinants**					
**Streptomycin Resistance Determinants**					
strA	strA-F	TTGATGTGGTGTCCCGCAATGC	383	57	(Hochhut et al., 2001)
strA	strA-R	CCAATCGCAGATAGAAGGCAA			(Hochhut et al., 2001)
strB	strB-F	CCGCGATAGCTAGATCGCGTT	515	60.5	(Ramachandran et al., 2007)
strB	strB-R	CGACTACCAGGCGACCGAAAT			(Ramachandran et al., 2007)
aadA1-like	aadA1a-F	GTGGATGGCGGCCTGAAGCC	526	70	(Sandvang et al., 1997)
aadA1-like	aadA1a-B	ATTGCCCAGTCGGCAGCG			(Sandvang et al., 1997)
aadA2	aadA2-F	TGTTGGTTACTGTGGCCGTA	622	60	(Walker et al., 2001)
aadA2	aadA2-B	GATCTCGCCTTTCACAAAGC			(Walker et al., 2001)
rpsL of *V. cholerae*	Vc-rpsL-F[a]	GAATTTTGCGTCCCTATTTGTG	500	60	This study
rpsL of *V. cholerae*	Vc-rpsL-R[a]	GGCCTTACTTAACGCTTCTC			This study
rpsL of *V. vulnificus*	Vv-rpsL-F[a]	TTGCGTGGTTGGGGATTAG	480	60	This study
rpsL of *V. vulnificus*	Vv-rpsL-R[a]	AGTGTTTGGCCTTACTTAACG			This study
**Class A Carbapenemases**					
IMI1-3, NMC-A	IMI(NMC)-F1	TGCGGTCGATTGGAGATAAA	399	50	(Hong et al., 2012)
	IMI(NMC)-R1	CGATTCTTGAAGCTTCTGCG			(Hong et al., 2012)
SME1-3	SME-F1	ACTTTGATGGGAGGATTGGC	551		(Hong et al., 2012)
	SME-R1	ACGAATTCGAGCATCACCAG			(Hong et al., 2012)
NMC-A	NMC1	GCATTGATATACCTTTAGCAGAGA	2,158	50	(Radice et al., 2004)
	NMC4	CGGTGATAAAATCACACTGAGCATA			(Radice et al., 2004)
SME	IRS-5	AGATAGTAAATTTTATAG	1,138	50	(Queenan et al., 2000)
	IRS-6	CTCTAACGCTAATAG			(Queenan et al., 2000)

IMI	IMI-A	ATAGCCATCCTTGTTTAGCTC	818	50	(Aubron et al., 2005)
	IMI-B	TCTGCGATTACTTTATCCTC			(Aubron et al., 2005)
KPC 1-5	multi KPC-F	CATTCAAGGGCTTTCTTGCTGC	538	56	(Dallenne et al., 2010)
	multi KPC-R	ACGACGGCATAGTCATTTGC			(Dallenne et al., 2010)
**Class B carbapenemases**					
IMP variants except IMP-9, IMP-16, IMP-18, IMP-22 and IMP-25	multi IMP F	TTGACACTCCATTTACDG	139	56	(Dallenne et al., 2010)
	multi IMP R	GATYGAGAATTAAGCCACYCT			(Dallenne et al., 2010)
VIM variants including VIM-1 and VIM-2	multi VIM 1-2 F	GATGGTGTTTGGTCGCATA	390		(Dallenne et al., 2010)
	multi VIM 1-2 R	CGAATGCGCAGCACCAG			(Dallenne et al., 2010)
NDM-1	NDM-F	GGTTTGGCGATCTGGTTTTC	621	52	(Poirel et al., 2011)
	NDM-R	CGGAATGGCTCATCACGATC			(Poirel et al., 2011)
**OXA-carbapenemases (class D)**					
OXA-48-like	multi OXA-48 F	GCTTGATCGCCCTCGATT	281	56	(Dallenne et al., 2010)
	multi OXA-48 R	GATTTGCTCCGTGGCCGAAA			(Dallenne et al., 2010)
**AmpC β-Lactamases**					
ACC	ACC F	AACAGCCTCAGCAGCCGGTTA	346	64	(Perez-Perez and Hanson, 2002)
	ACC B	TTCGCCGCAATCATCCCTAGC			(Perez-Perez and Hanson, 2002)
LAT-1-4, CMY-2-7, BIL-1,	CIT F	TGGCCAGAACTGACAGGCAAA	462		(Perez-Perez and Hanson, 2002)
	CIT B	TTTCTCCTGAACGTGGCTGGC			(Perez-Perez and Hanson, 2002)
DHA-1, DHA-2	DHA F	AACTTTCACAGGTGTGCTGGGT	405		(Perez-Perez and Hanson, 2002)
	DHA B	CCGTACGCATACTGGCTTTGC			(Perez-Perez and Hanson, 2002)
MIR-1T, ACT-1	EBC F	TCGGTAAAGCCGATGTTGCGG	302		(Perez-Perez and Hanson, 2002)
	EBC B	CTTCCACTGCGGCTGCCAGTT			(Perez-Perez and Hanson, 2002)
FOX-1-5b	FOX F	AACATGGGGTATCAGGGAGATG	190		(Perez-Perez and Hanson, 2002)
	FOX B	CAAAGCGCGTAACCGGATTGG			(Perez-Perez and Hanson, 2002)
MOX-1, MOX-2, CMY-1,CMY-8-11	MOX F	GCTGCTCAAGGAGCACAGGAT	520		(Perez-Perez and Hanson, 2002)
	MOX B	CACATTGACATAGGTGTGGTG			(Perez-Perez and Hanson, 2002)
**Other β-Lactamases**					
bla$_{OXA\text{-}1like}$	OXA1-F	AGCAGCGCCAGTGCATCA	708	59	(Guerra et al., 2000)
bla$_{OXA\text{-}1like}$	OXA1-B	ATTCGACCCCAAGTTTCC			(Guerra et al., 2000)
bla$_{pse1}$	pse1-F	CGCTTCCCGTTAACAAGTAC	419	65	(Sandvang et al., 1997)
bla$_{pse1}$	pse1-B	CTGGTTCATTTCAGATAGCG			(Sandvang et al., 1997)
bla$_{tem1\ like}$	OT-1	TTGGGTGCACGAGTGGGT	503	55	(Arlet and Philippon, 1991)
bla$_{tem1\ like}$	OT-2	TAATTGTTGCCGGGAAGC			(Arlet and Philippon, 1991)

**Integrons**					
intI1, class 1 integron	Int1F	CCTGCACGGTTCGAATG	497	59	(Kitiyodom et al., 2010)
intI1, class 1 integron	Int1R	TCGTTTGTTCGCCCAGC			(Kitiyodom et al., 2010)

T_a annealing temperature;

[a] used for amplification and sequencing

References

Andrews, J.M. (2009). BSAC standardized disc susceptibility testing method (version 8). *J Antimicrob Chemother* 64**,** 454-489. doi: 10.1093/jac/dkp244.

Arlet, G., and Philippon, A. (1991). Construction by polymerase chain reaction and use of intragenic DNA probes for three main types of transferable beta-lactamases (TEM, SHV, CARB) [corrected]. *FEMS Microbiol Lett* 66**,** 19-25.

Aubron, C., Poirel, L., Ash, R.J., and Nordmann, P. (2005). Carbapenemase-producing Enterobacteriaceae, U.S. rivers. *Emerg Infect Dis* 11**,** 260-264. doi: 10.3201/eid1102.030684.

Bauer, A., and Roervik, L.M. (2007). A novel multiplex PCR for the identification of *Vibrio parahaemolyticus, Vibrio cholerae* and *Vibrio vulnificus*. *Letters in Applied Microbiology* 45**,** 371-375.

CLSI (2010a). Clinical and Laboratory Standards Institute. *Methods for Antimicrobial Dilution and Disk Susceptibility Testing of Infrequently Isolated or Fastidious Bacteria; Approved Guideline —Second Edition M45-A2.* CLSI, Wayne, PA, USA, 2010.

CLSI (2010b). Clinical and Laboratory Standards Institute. *Performance Standards for Antimicrobial Susceptibility Testing; Twentieth Informational Supplement M100-S20.* CLSI, Wayne, PA, USA, 2010.

CLSI (2010c). Clinical and Laboratory Standards Institute. *Performance Standards for Antimicrobial Susceptibility Testing; Twentieth Informational Supplement M100-S20-U.* CLSI, Wayne, PA, USA, 2010.

CLSI (2013). Clinical and Laboratory Standards Institute. *Performance Standards for Antimicrobial Disk and Dilution Susceptibility Tests for Bacteria Isolated From Animals; Second Informational Supplement VET01-S2*. CLSI, Wayne, PA, USA, 2013.

CLSI (2015). Clinical and Laboratory Standards Institute. *Performance Standards for Antimicrobial Susceptibility Testing; Twenty-fifth Informational Supplement M100-S25*. CLSI, Wayne, PA, USA, 2015.

Dallenne, C., Da Costa, A., Decre, D., Favier, C., and Arlet, G. (2010). Development of a set of multiplex PCR assays for the detection of genes encoding important beta-lactamases in Enterobacteriaceae. *J Antimicrob Chemother* 65**,** 490-495. doi: 10.1093/jac/dkp498.

Eucast (2015). *The European Committee on Antimicrobial Susceptibility Testing. Breakpoint tables for interpretation of MICs and zone diameters. Version 5.0.* [Online]. Available: http://www.eucast.org [Accessed].

Guerra, B., Soto, S., Cal, S., and Mendoza, M.C. (2000). Antimicrobial resistance and spread of class 1 integrons among Salmonella serotypes. *Antimicrob Agents Chemother* 44**,** 2166-2169.

Hochhut, B., Lotfi, Y., Mazel, D., Faruque, S.M., Woodgate, R., and Waldor, M.K. (2001). Molecular analysis of antibiotic resistance gene clusters in Vibrio cholerae O139 and O1 SXT constins. *Antimicrobial Agents and Chemotherapy* 45**,** 2991-3000.

Hong, S.S., Kim, K., Huh, J.Y., Jung, B., Kang, M.S., and Hong, S.G. (2012). Multiplex PCR for rapid detection of genes encoding class A carbapenemases. *Ann Lab Med* 32**,** 359-361. doi: 10.3343/alm.2012.32.5.359.

Hoshino, K., Yamasaki, S., Mukhopadhyay, A.K., Chakraborty, S., Basu, A., Bhattacharya, S.K., Nair, G.B., Shimada, T., and Takeda, Y. (1998). Development and evaluation of a multiplex PCR assay for rapid detection of toxigenic Vibrio cholerae O1 and O139. *FEMS Immunology and Medical Microbiology* 20**,** 201-207.

Kitiyodom, S., Khemtong, S., Wongtavatchai, J., and Chuanchuen, R. (2010). Characterization of antibiotic resistance in Vibrio spp. isolated from farmed marine shrimps (Penaeus monodon). *FEMS Microbiology Ecology* 72**,** 219-227.

National Food Institute, T.U.O.D. (2013). DANMAP 2013. Use of antimicrobial agents and occurrence of antimicrobial resistance in bacteria from food animals, food and humans in Denmark. .

Perez-Perez, F.J., and Hanson, N.D. (2002). Detection of plasmid-mediated AmpC beta-lactamase genes in clinical isolates by using multiplex PCR. *J Clin Microbiol* 40**,** 2153-2162.

Poirel, L., Walsh, T.R., Cuvillier, V., and Nordmann, P. (2011). Multiplex PCR for detection of acquired carbapenemase genes. *Diagn Microbiol Infect Dis* 70**,** 119-123. doi: 10.1016/j.diagmicrobio.2010.12.002.

Queenan, A.M., Torres-Viera, C., Gold, H.S., Carmeli, Y., Eliopoulos, G.M., Moellering, R.C., Jr., Quinn, J.P., Hindler, J., Medeiros, A.A., and Bush, K. (2000). SME-type carbapenem-hydrolyzing class A beta-lactamases from geographically diverse Serratia marcescens strains. *Antimicrob Agents Chemother* 44**,** 3035-3039.

Radice, M., Power, P., Gutkind, G., Fernandez, K., Vay, C., Famiglietti, A., Ricover, N., and Ayala, J.A. (2004). First class a carbapenemase isolated from enterobacteriaceae in Argentina. *Antimicrob Agents Chemother* 48**,** 1068-1069.

Ramachandran, D., Bhanumathi, R., and Singh, D.V. (2007). Multiplex PCR for detection of antibiotic resistance genes and the SXT element: application in the characterization of Vibrio cholerae. *J Med Microbiol* 56**,** 346-351. doi: 10.1099/jmm.0.46655-0.

Sandvang, D., Aarestrup, F.M., and Jensen, L.B. (1997). Characterisation of integrons and antibiotic resistance genes in Danish multiresistant Salmonella enterica Typhimurium DT104. *FEMS Microbiol Lett* 157**,** 177-181.

Shaw, K.S., Rosenberg Goldstein, R.E., He, X., Jacobs, J.M., Crump, B.C., and Sapkota, A.R. (2014). Antimicrobial susceptibility of *Vibrio vulnificus* and *Vibrio parahaemolyticus* recovered from recreational and commercial areas of Chesapeake Bay and Maryland Coastal Bays. *PLoS One* 9**,** e89616. doi: 10.1371/journal.pone.0089616.

Shirai, H., Nishibuchi, M., Ramamurthy, T., Bhattacharya, S.K., Pal, S.C., and Takeda, Y. (1991). Polymerase chain reaction for detection of the cholera enterotoxin operon of Vibrio cholerae. *J Clin Microbiol* 29**,** 2517-2521.

Walker, R.A., Lindsay, E., Woodward, M.J., Ward, L.R., and Threlfall, E.J. (2001). Variation in clonality and antibiotic-resistance genes among multiresistant Salmonella enterica serotype typhimurium phage-type U302 (MR U302) from humans, animals, and foods. *Microb Drug Resist* 7**,** 13-21. doi: 10.1089/107662901750152701.
